# Sex Identification and Species Confirmation in Modern and Archeological Caprine Enamel

**DOI:** 10.1021/acs.jproteome.5c00012

**Published:** 2025-08-06

**Authors:** Paula Kotli, David Morgenstern, Shifra Ben-Dor, Liora Kolska Horwitz, Elisabetta Boaretto

**Affiliations:** † Scientific Archaeology and D-REAMS Radiocarbon Dating Laboratory, 34976 Weizmann Institute of Science, 760001 Rehovot, Israel; ‡ Nancy and Stephen Grand Israel National Center for Personalized Medicine G-INCPM, Weizmann Institute of Science, 760001 Rehovot, Israel; § Bioinformatics Unit, Life Sciences Core Facilities, Weizmann Institute of Science, Rehovot 760001, Israel; ∥ National Natural History Collections, E. Safra-Givat Ram Campus, 26742 The Hebrew University of Jerusalem, 96194 Jerusalem, Israel

**Keywords:** paleoproteomics, PRM mass spectrometry assay, enamelin, AmelX, AmelY, zooarcheology

## Abstract

Proteomics has become a transformative tool for species and sex determination. This study introduces a novel methodology that integrates amelogenin (Amel) and enamelin (Enam) proteins extracted from the tooth enamel of caprines. Since morphologically, osteological remains of sheep and goats often cannot be easily discriminated, we developed our method on both modern domestic sheep (*Ovis aries*) and goats (*Capra hircus*) to establish unique proteomic signatures for each species for sex and species identification. Applying a targeted parallel reaction monitoring (PRM) assay, we validated the sex and species of 8 modern domestic sheep and 6 domestic goats. We then applied the same method to 10 ancient samples dating to the early eighth millennium BC Neolithic period. For sex determination, AmelY peptides were exclusively detected in modern male samples, while AmelX peptides were present in both sexes. Sex determination in 10 Neolithic samples demonstrated 40% males. For species determination, Enam species-specific peptides with single amino acid variations (SAAVs) successfully distinguished the modern caprine species. In the 10 archeological samples, only goat-specific Enam peptides were detected, validating previous zooarcheological results for this assemblage using morphology and mtDNA analysis. Robust peptide intensities and strong statistical correlations between modern and ancient data sets confirm the preservation of these unique markers in caprine enamel, expanding the application of proteomics to modern, archeological, and paleontological samples.

## Graphical abstract

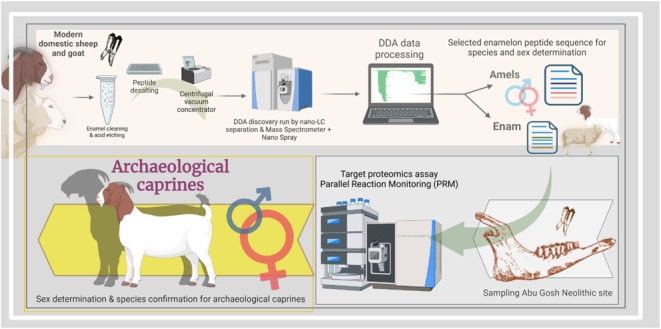

## Introduction

The study of ancient biomolecules has transformed our understanding of the past, with paleoproteomics emerging as a valuable tool in archeological research for species and sex determination.^1–5^ Recent advances in techniques such as Zooarcheology by Mass Spectrometry (ZooMS) have revolutionized identification of animal species from even small bone and tooth fragments, expanding our ability to recognize species across a variety of zooarcheological finds.^6,7^ ZooMS enables species identification by targeting collagen peptides in bone and dentin, but it relies heavily on good preservation of organic material in these tissues.^8–18^ This dependency can be a limiting factor as bone collagen is often degraded in ancient samples and may not always yield sufficient organic material for analysis.^19^

In contrast, enamel is the most stable mineralized tissue in the vertebrate body and so offers unique advantages for biomolecular preservation.^20^ During enamel formation (amelogenesis), the matrix initially comprises around 30% mineral by weight with the remainder consisting of water and organic material. The main structural proteins of the enamel proteome are secreted by ameloblasts and comprise amelogenin (Amels or AmelX and AmelY), enamelin (Enam), ameloblastin (Ambn), amelotin (Amtn), tuftelin (Tuft1), and the proteinases: matrix metalloproteinase-20 (MMP-20 or enamelysin) and kallikrein-4 (KLK-4).^21^ As the enamel matures, the bioapatite crystals grow, while organic material and pore fluid are gradually reduced to approximately 1 and 4%, respectively, producing a highly stable structure that can resist most diagenetic processes^22–26^ (although exposure to heat or extended burial can, in some circumstances, affect the organic matrix of enamel^1,3,27,28^ ). Thus, even under adverse environmental conditions, enamel often retains small quantities of preserved proteins within its mineral matrix, which makes it a reliable substrate for proteomic studies of ancient specimens.^3,29–32^

AmelX and AmelY, paralogs of amelogenin, are found on the X and Y chromosomes, respectively, making it possible to identify sex using DNA amplification and further sequencing.^33–37^ The sex-specific sequence variability of the amelogenin gene has been widely applied for sex determination in a variety of animal taxa, using only soft tissue sequences.^38–42^ Only in recent years have researchers successfully extracted the proteins found in tooth enamel to determine sex in humans^43–48^ as well as in ancient samples of extinct and extant hominids.^28,49–52^ Cappellini et al.^1^ used proteins extracted from tooth enamel proteome (Ambn, AmelX, Enam, Amtn, MMP-20), as well as collagen and other nonspecific enamel proteins, to investigate fossil rhinoceros taxonomy, opening the door to taxonomic studies of fauna in samples with poorly preserved collagen.^1,3^ More recently, Kotli et al.^53^ developed a method for sex determination for modern and ancient cattle (*Bos* sp.) tooth enamel, based on label-free quantification (LFQ). The authors developed the method using known-sexed modern domestic cattle and then, based on sex identification on two unique AmelY peptides, applied this method to *Bos* samples of unknown sex from a Neolithic site (dated to the second half of the eighth millennium BC–first half of the seventh millennium BC). Subsequently, other researchers have also determined the sex of modern and ancient ungulates based on amelogenin extracted from enamel, attesting to promising developments in enamel proteomics for detecting sex-specific peptide markers.^4,5,53^ However, challenges persist, particularly with ancient samples where diagenetic processes introduce variability in post translational modifications (PTMs) and may fragment peptides, complicating their identification and quantification.^2,3^

In the context of the domestic caprines studied here, sheep (*Ovis aries*) and goats (*Capra hircus*), the problem of diagenesis is compounded by the limited availability of reference data for unique peptide markers in caprines that are essential for distinguishing between these closely related species. We deemed that an approach targeting unique peptides was essential to overcome the inherent challenges posed by diagenetic changes in the ancient samples. Therefore, for this study, we developed a precise list of target peptides using data-dependent acquisition (DDA) for initial identification. This was followed by parallel reaction monitoring (PRM) mass spectrometry assay for accuracy, tailored to address the distinct challenges of sex and species determination in modern caprine enamel. As a test case, we analyzed archeological caprine samples in order to confirm previous species identifications of goat, that had been undertaken on the same assemblage using standard zooarcheological methods as well as mtDNA analysis.^54,55^

The robust preservation and excellent taxonomic resolution of enamelin (ENAM) peptides in fossil enamel has been demonstrated across multiple deep-time contexts. Bray et al.^56^ showed that ENAM peptides can distinguish bovid taxa up to 120,000 years old, while Green et al.^57^ extended this to over one million years in African megafauna, including hippopotamids and proboscideans. Most recently, Paterson et al.^58^ successfully retrieved informative ENAM peptides from an early Miocene rhinocerotid molar dated to ∼19.9 ± 0.2 Ma, using them to resolve its phylogenetic placement within Ceratotheriinae. Together, these studies establish ENAM as a remarkably durable and phylogenetically informative biomolecule, supporting its use in our assay to differentiate closely related caprines in both modern and archeological contexts.

By focusing on single amino acid variations (SAAVs) within amelogenin (Amel) and enamelin (Enam), we established a dual-marker approach with Amel peptides facilitating sex identification and Enam peptides serving as reliable species-specific markers to differentiate sheep from goats. Amelogenin has commonly been used for sex identification, as AmelX or AmelY gene copies of amelogenin are found on the X and Y chromosomes, respectively, making it possible to identify sex with the use of DNA amplification and further sequencing.^39,59^ Furthermore, our preliminary alignment of enamelin suggested that this protein has the potential to be used for taxonomic determination between caprines. Enam is one of the six common proteins in tooth enamel and occurs in a low concentration in mature enamel but has the longest sequence of all enamel proteins in humans, encoded by 1142 amino acids and a signal peptide of 39 amino acids.^60^ In the Enam sequence for sheep, position 187 is occupied by lysine (Lys) and 212 by phenylalanine (Phe), while in the same positions in the goat Enam sequence, arginine (Arg) and tyrosine (Try), respectively, occupied these positions.

## Materials and Methods

### Modern Samples

Eleven caprine teeth were extracted from complete lower jaws (mandibles) of eight modern domestic sheep (*O. aries*)—five females and three males, and three domestic goats (*C. hircus*)—all male (for more details, see photographs in Supporting Information (SI) Figures 1–3). The specimens were sourced from a local slaughterhouse, specifically for research purposes. Additionally, we analyzed mandible teeth of three modern female goats that had been donated to the Israeli National Veterinary Center (Beit Dagan) for research purposes, and are currently curated in the osteological collection of the Kimmel Center for Archaeological Science (Weizmann Institute); see SI Figure 4. All samples were taken from animals of known species and sex (see Table 1). Individual teeth were extracted from the jaws, cleaned, and labeled for storage at 4 °C. Note: Samples WIS 304 and WIS 305 represent repeated analyses of the same female goat specimen for reproducibility.

**Table 1:** Modern Caprine Sample Information Including the Laboratory Sample Number, Proteomic Batch Number, Laboratory Animal ID Number, Origin, Animal Species, Animal Tag Number from Abattoir, Sex Listed in Abattoir Documentation, and Mandible Tooth Type (M = Molar; PM = Premolar; I = Incisor)^a^

sample number	proteomic batch	animal number WIS	origin	species	animal tags ID numbers	sex listed in Abattoir	tooth type
430	EB22325	24.1.001	Shefa-Amr	*O. aries*	9225; 4805328	female	I
431	EB22325	24.1.002	Shefa-Amr	*O. aries*	1054; 4743873	female	I
432	EB22325	24.1.003	Shefa-Amr	*O. aries*	3084; 5200324	female	I
433	EB22325	24.1.004	Shefa-Amr	*O. aries*	9610; 4938425	female	I
434	EB22325	24.1.005	Shefa-Amr	*O. aries*	2895; 5199231	female	I
436	EB22325	24.2.001	Shefa-Amr	*O. aries*	0548; 5399124	male	I
437	EB22325	24.2.002	Shefa-Amr	*O. aries*	0423; 5399236	male	I
438	EB22325	24.2.003	Shefa-Amr	*O. aries*	0567; 5399266	male	I
441	EB22325	24.3.001	Shefa-Amr	*C. hircus*		male	I
442	EB22325	24.3.002	Shefa-Amr	*C. hircus*		male	I
444	EB22325	24.3.004	Shefa-Amr	*C. hircus*		male	I
302	EB22325	Capra hircus 7	WIS Collection	*C. hircus*		female	I
303	EB22325	Capra hircus 8	WIS Collection	*C. hircus*		female	I
304	EB22325	Capra hircus 4	WIS Collection	*C. hircus*		female	PM
305	EB22325	Capra hircus 4	WIS Collection	*C. hircus*		female	M

a Samples WIS 304 and WIS 305 are from the same female goat specimen; the two samples served as a repeat control.

### Archeological Samples

#### Abu Gosh (Map Ref NIG 2105/6345)

The archeological site of Abu Gosh is situated in the Judean Hills, within the present-day village of Abu Gosh, approximately 12 km west of Jerusalem (Israel), at the intersection of the Mediterranean and Irano-Turanian phytogeographic zones.^55,61,62^ Initial test excavations at the site took place in 1950 (under the direction of Jean Perrot), followed by another round of excavations in 1967, under the direction of Lechevallier.^61^ Both investigations uncovered remains of a sedentary mid-Pre-Pottery Neolithic B (MPPNB) settlement dating back to the early part of the eighth millennium BC. Finds recovered comprised architectural elements (such as stone-built structures with plaster floors), worked flint artifacts, including typical MPPNB tools such as sickle blades and arrowheads, human burials (including plaster skulls), and a large assemblage of faunal remains. In 1995, further excavations at the site (directed by Hamoudi Khalaily and Ofer Marder), revealed a topmost Pottery Neolithic layer, beneath which lay two Mid-PPNB layers (III and IV).^63^

The enamel samples used in this study all derive from caprine teeth recovered during the Lechevallier excavation and date to the MPPNB.^61^ These remains were identified as goat using standard zooarcheological procedures^55^ including comparison with a modern osteological collection, aided by osteological guides detailing morphological traits specific to sheep and goats.^54,64,65^ In addition, mtDNA analysis confirmed the presence of goats. To avoid as much as much as possible sampling of the same animal, we chose the same dental element deriving from the same side of the jaw, namely, lower premolars, as well as teeth originating from archeological contexts that were distinct and physically distant from each other. For full details of the samples, see Table 2 and also sample photographs in SI Figures 5–7.

**Table 2:** Information for Archeological Caprine Samples Including the Sample Number, Sample Locus, and Square in the Archeological Site, Animal Species as Identified by Zooarcheologists, Animal Species as Identified by Enam Sequences (This Research Study), Sex by Amelogenin (This Research Study), Tooth Type (PM = Premolar, M = Molar), and Period (MPPNB = Mid-Pre-Pottery Neolithic B)

sample number	proteomic batch	Loc. Sq. (location square)	species	species id zooarcheology	species ID Enam	sex ID Amel	tooth type	period
401	EB22325	AG B1328 (2245 B)	*Caprine*	*Capra*	*Capra*	male	M	MPPNB
420	EB22325	AG70-BYD17; Z1B07	*Caprine*	*Capra*	*Capra*	female	PM	MPPNB
421	EB22325	AG346 AZ3752	*Caprine*	*Capra*	*Capra*	male	PM	MPPNB
422	EB22325	AG70 394-1 AZ7156	*Caprine*	*Capra*	*Capra*	female	PM	MPPNB
423	EB22325	AG343 AZ3554	*Caprine*	*Capra*	*Capra*	female	PM	MPPNB
424	EB22325	AG165 AZ280	*Caprine*	*Capra*	*Capra*	male	PM	MPPNB
425	EB22325	AG70 938-45 AZ7082	*Caprine*	*Capra*	*Capra*	male	PM	MPPNB
426	EB22325	AG76 832-24 AZ6934 AZ6 132-24	*Caprine*	*Capra*	*Capra*	female	PM	MPPNB
427	EB22325	AG70AZ6503 892-32	*Caprine*	*Capra*	*Capra*	female	PM	MPPNB
428	EB22325	AG705 AZ5548	*Caprine*	*Capra*	*Capra*	female	PM	MPPNB

### Samples Preparation and Cleaning

Each tooth (modern and archeological) was first cleaned mechanically using soft brushes and cold DDW water (see also SI Figures 8–11) to exclude any extraneous material (e.g., sediment, organic material). Further cleaning was performed using a mechanical dremel drill to remove external calculus and internal dentin (full process photographs; see SI Figure 12). An enamel fragment (10 mm × 15 mm) was then cut out and extracted. A final examination of each enamel sample was carried out using a Nikon SMZ800N binocular microscope to ensure it was clean with no extraneous material adhering. Finally, etching was performed on the extracted fragment under a chemical hood.

### Sample Preparation for MS

The extracted and clean piece of caprine enamel (20–50 mg) was immersed for 30 s in 3% H_2_O_2_, rinsed with DDW and the solution discarded. The enamel piece was then etched for 2 min in freshly prepared 5% HCl. This solution was discarded. A second etching step in 200–500 μL 5% HCl (to a final enamel concentration of 10 mg/100 μL 5% HCl) allowed the enamel fragments to completely dissolve at room temperature (no more than 90 min), and the solution was retained on ice. The samples were frozen and stored at −80 ^*°*^C for desalting.

### Mass Spectrometry

Dissolved samples were desalted using an Oasis HLB 96 well plate (Waters), following the manufacturer’s instructions; samples were loaded into the wells using vacuum pull, followed by three washes of 300 μL 0.1% TFA. The peptides were then eluted by passing 50 μL of 50% acetonitrile and 0.1% FA. The resulting peptides were loaded onto, and separated on a 50 cm uPAC Neo, reversed-phase C18 column (Thermo Fisher), mounted on a nanoAquity (Waters) nanoLC instrument. Peptides were eluted from the column using a 50 min gradient from 4–30% B (99.9% acetonitrile and 0.1% formic acid) at 500 nL/min flow. The peptides were eluted into a Tribrid Fusion Lumos mass spectrometer (Thermo Fisher) using a FlexIon nano-ESI source, through a 20 μm ID emitter (Fossil IonTech, Madrid) at 2.8 kV. Blank injections interspaced samples to allow washing of peptide carryover. Data were acquired as either DDA or PRM methods. DDA: MS1 resolution was set to 120,000@ 200 *m*/*z*, at a mass range of 375–1650 *m*/*z*, with maximum injection time set to 246 ms, and AGC set to 100%. MS2 data were acquired via a Top Speed, 3s method. MIPS was set to peptide, dynamic exclusion set to 30 s, charge state filtering to 2–8, and intensity threshold to 5 × 10^4^. The isolation window was set to 1 *m/z*, and fragmentation was set to HCD with 30 NCE; first mass was set to 130 m/z; injection time to Auto and AGC target to 100 PRM; the PRM method included MS1 scan data in one experiment and the tMS2 experiment. MS1 scans were set to the same parameters as those of the DDA method above. PRM was set to minimum 5 points across the peak, 1 m/z isolation window, AGC set to 100%, injection time set to Auto, resolution at 15 K @ 200 m/z, HCD fragmentation set to 30 NCE at loop time of 2 s. Selected precursors for fragmentation are given in Table 3.

**Table 3:** Unique Enam and Amel Peptide Sequence Data with m/z Values and Charge States

peptide	*m*/*z*	*z*
R IPPGFGRPPG	575.8276	2
K IPPGFGRPPG	561.8246	2
PFFGYFGF H	559.7584	2
IRHPYPSY	516.7667	2
SmIRHPYP	508.7527	2
IPHR IPPGFGRPPG	499.9528	3
IPHK IPPGFGRPPG	490.6174	3
mLRYPYP	478.2389	2
LRYPYP	404.7212	2
IRHPYP	391.719	2

### Bioinformatics Analysis

The sheep and goat genomes available in standard databases in March 2022 lacked chromosome Y. We predicted the sequences from domestic breeds whose genomes were available: *O. aries* and East Friesien breed EF_391 (NCBI BioSample: SAMN18719720), and *C. hircus* Saanen dairy goat breed (SAMN14408556). Amelx and Amely were manually constructed from the genome sequences. The full alignments of Figure 3 are available in SI Figure 13. To strengthen the finding, additional sheep Amel genes were defined in October 2022, from male genomes of different breeds: Kermani (SAMN23436134); Dorper (SAMN18719831); Romanov x Dorper (SAMN19573257); Hu (SAMN13678651). No additional male goat genomes were available at that time. A full alignment of various sheep genome sequences is available in SI Figure 14. The full putative coding sequences and proteins of all goat and sheep species are provided in the Supporting Data. Alignments were performed with Muscle 3.8.31.^66^

### Data Analysis

Amelogenin sequences were based on published Uniprot sequences and modified in-house based on online genomic data that was tailored. A data search was undertaken using a Byonic search engine (Protein Metrics Inc.) with a database that contained the peptides listed above with the relevant modifications (oxidation on M). MS1 tolerance was set to 10 ppm, while MS2 was set to 20 ppm. Data was filtered using a Byonic target-decoy method set to 1% FDR and inspected manually. Quantification was performed using Skyline software (version 23.1.0.455) with MS2 transients. Byonic mzID files were imported into Skyline along with the RAW files based on the MS2 spectra. Bulk deamidation (in-house scripts) and statistical analysis were performed using RStudio version 2023.03.1 (registered under Posit Software), stats package, plots by in-house scripts.

## Results

### Selection of Suitable Peptides for the Development of a Parallel Reaction Monitoring (PRM) Assay

Modern and ancient enamel proteins can undergo physical alterations during the process of in vivo enamel maturation, diagenesis (for archeological samples), and/or acid extraction in the laboratory. As a result, unique peptide sequences or diagenetic peptide forms (“diagenetiforms”)^2^ are produced, altering the native *m*/*z* of the peptide sequences. To counter this problem, in our development of a targeted peptide mass spectrometry (MS) assay, we chose peptide sequences that were consistently identified by MS in both modern and ancient enamel. Our experimental design used data-dependent acquisition (DDA) runs of modern samples to identify potential peptides for future targeted analysis, and these were then used to confirm our results on ancient samples. We established the applicability of the DDA results on modern samples to parallel reaction monitoring (PRM) assays of archeological samples, demonstrating where the peptide identifications were identical. To this end, we first selected potential native peptide sequences from the DDA identification results of eight modern domestic sheep (*O. aries*) and seven modern goat (*C. hircus*) of known species and sex. As a case study to demonstrate the methods applicability to ancient goat samples, we ran ten enamel samples from the Neolithic archeological site of Abu Gosh (Tables 1 and 2, respectively).

Below, we provide the DDA results on which we based our selection of the target native peptide sequence for the later PRM assay, to provide a simple and accurate determination of sex and species.

#### Selection of Unique Amel Sequences for Sex Determination from Modern Samples

Only incomplete caprine amelogenin sequences were found in the public database that we checked (GenBank, March 2022). Consequently, we predicted and manually curated Amel sequences from genome data and generated full amelogenin amino acid sequences. This allowed us to identify and then build a unique list of native dimorphic Amel sequences from DDA data for modern caprines. From the final results of the MS2 identified PSMs, we chose AmelY peptide sequences containing SAAVs at positions Leu46 and Tyr48, specifically “ **L** R **Y** PYP” (AmelY;[M+2]^2+^ 404.7212 *m*/*z*) and “M­(ox) **L** R **Y** PYP” (AmelY;[M+2]^2+^ 478.2389 *m*/*z*). Kotli et al.^53^ already demonstrated that the unique sequence of the AmelY dimorphic peptide “ **L** R **Y** PYP” can be used to determine male sex in cattle. This unique AmelY peptide was also found to be present in the male sheep and male goat samples analyzed here (see Figure 1a.1,a.2 samples, chromatogram I), while we also found another unique AmelY peptide, “M­(ox) **L** R **Y** PYP” that can be used for sex determination (see Figure 1a.1,1a.2 samples, chromatogram II). This unique AmelY peptide sequence was found solely in male caprine samples at XIC intensities between 2.48 × 10^6^ and 8.31 × 10^6^ (see Figure 2, filled green and blue icons). In addition, we found three common AmelX dimorphic peptide sequences that carry SAAVs on Ser44, Ile46, and His48. Two of these sequences, “ **S** M­(ox) **I** R **H** PYP” (AmelX;[M+2]^2+^ 508.7527 *m*/*z*) and “ **I** R **H** PYPSY” (AmelX;[M+2]^2+^ 516.7676 *m*/*z*), have also been confirmed as present in cattle,^53^ while the present research found a third new peptide sequence in caprines, “ **I** R **H** PYP” (AmelX;[M+2]^2+^ 391.7191 *m*/*z*). In modern caprine samples, the unique AmelX peptides show XIC intensities up to 1.22 × 10^8^, reflecting the good quality of our enamel etching (see Figure 1a.1,a.2 for male caprine samples and 1b.1,b.2 for female caprine samples [chromatograms III–V] and Figure 2 blue and green empty icons showing modern sheep and goat female samples, respectively). In addition, Figure 3 provides a visualization of the Amel dimorphic peptide sequence selected for further target proteomic assay. It is important to note that for all modern samples analyzed, only one sample (WIS 303) exhibited a low intensity (XIC of 9.14 × 10^4^) of AmelX dimorphic peptides, and only in one peptide “ **I** R **H** PYP”. The other two sequences show an XIC intensity above 5.23 × 10^6^.

**Figure 1:**
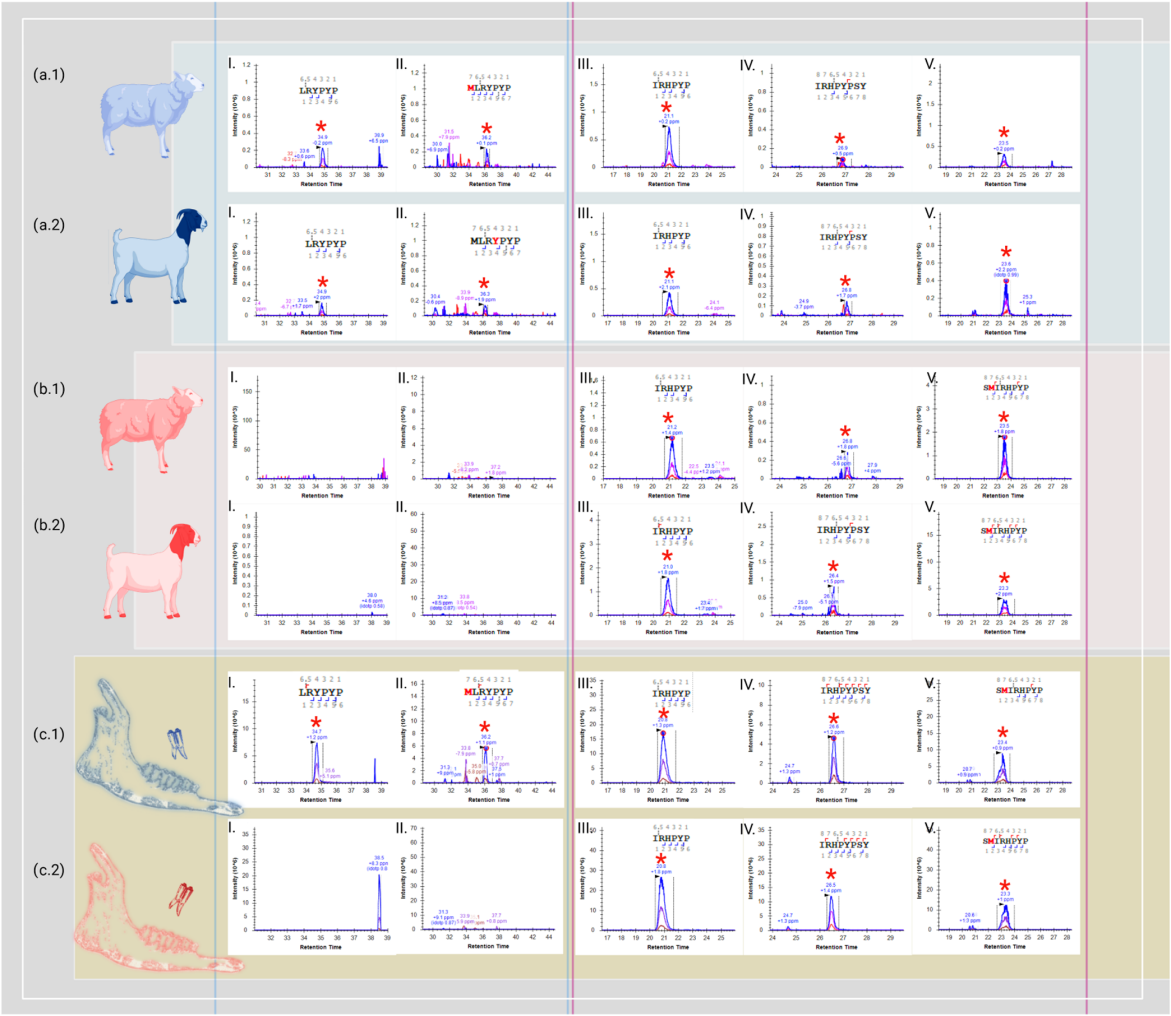
Presentation of XIC intensities of the isotopic envelope precursor obtained using Skyline software for modern samples: male sheep (a.1), male goat (a.2), female sheep (b.1), and female goat (b.2) for two unique AmelY and three unique AmelX peptides. In addition, two archeological samples from Abu Gosh (c.1 and c.2) were included. Unique AmelY peptide chromatograms found only in male samples (I) “ **L** R **Y** PYP” (AmelY;[M+2]^+2^ 404.7212 *m*/*z*) and (II) “M­(ox) **L** R **Y** PYP” (AmelY;[M+2]^+2^ 478.2389 *m*/*z*). AmelX unique peptides chromatograms: (III) “ **I** R **H** PYP” (AmelX;[M+2]^+2^ 391.7191 *m*/*z*), (IV) “ **I** R **H** PYPSY” (AmelX;[M+2]^+2^ 516.7676 *m*/*z*) and “ **S** M­(ox) **I** R **H** PYP” (AmelX;[M+2]^+2^ 508.7527 *m*/*z*). As anticipated from earlier Byonic search identifications, the retention time was as expected even in samples that did not undergo an MS/MS ID. AmelY peptides were detected solely in male samples, with none present in female samples. Conversely, all three distinct AmelX peptides appeared in both the male and female samples.

**Figure 2:**
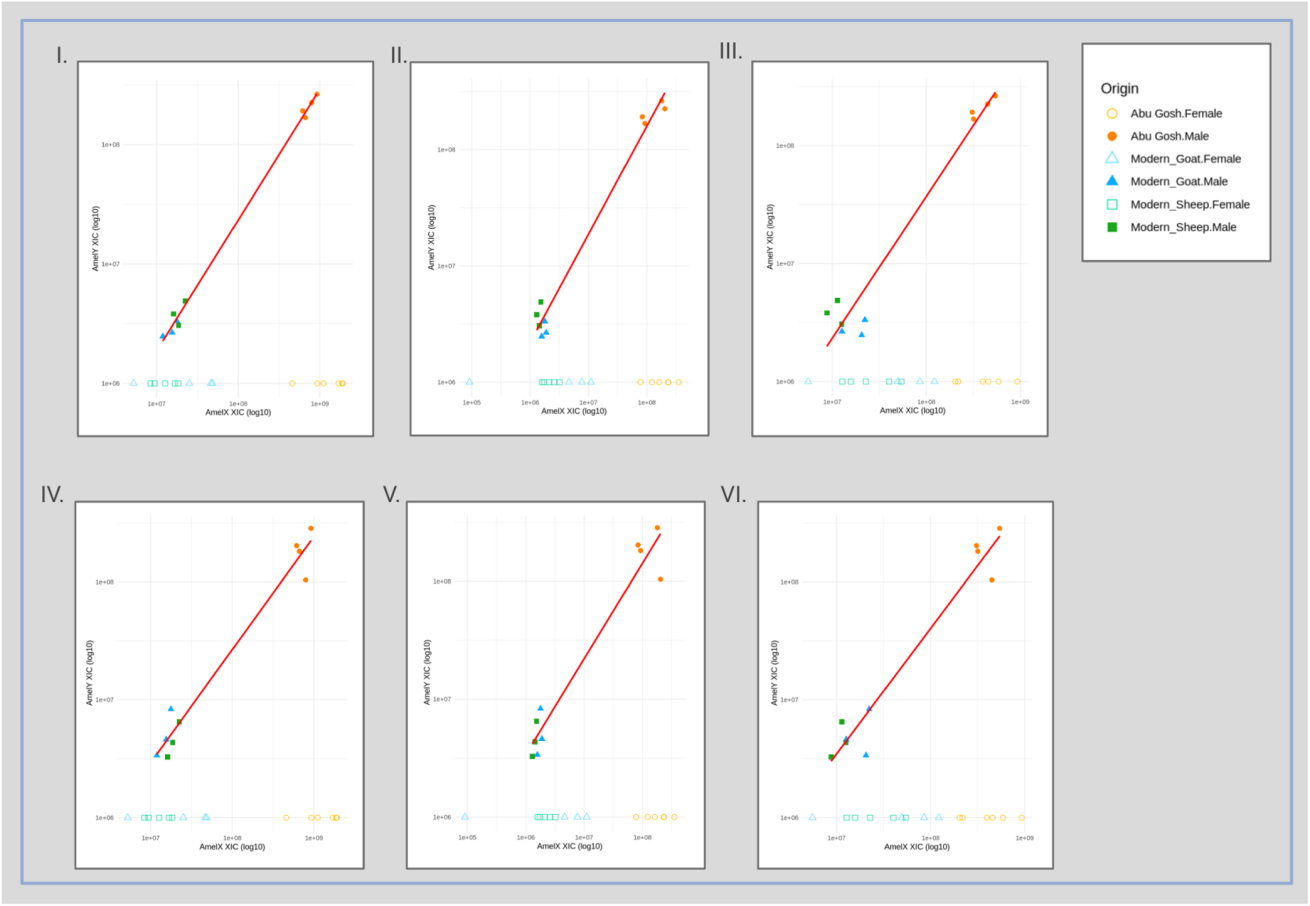
Each plot shows the relationship of ion intensities between different peptide pairs by ion intensities of AmelY versus AmelX unique native peptide intensities (I) “ **L** R **Y** PYP” (AmelY;[M+2]^+2^ 404.7212 *m*/*z*) vs “ **I** R **H** PYP” (AmelX;[M+2]^+2^ 391.7191 *m*/*z*); (II) “ **L** R **Y** PYP” (AmelY;[M+2]^+2^ 404.7212 *m*/*z*) vs “ **I** R **H** PYPSY” (AmelX;[M+2]^+2^ 516.7676 *m*/*z*); (III) “ **L** R **Y** PYP” (AmelY;[M+2]^+2^ 404.7212 *m*/*z*) vs “ **S** M­(ox) **I** R **H** PYP” (AmelX;[M+2]^+2^ 508.7527 *m*/*z*); (IV) “M­(ox) **L** R **Y** PYP” (AmelY;[M+2]^+2^ 478.2389 *m*/*z*) vs “ **I** R **H** PYP” (AmelX;[M+2]^+2^ 391.7191 *m*/*z*); (V) “M­(ox) **L** R **Y** PYP” (AmelY;[M+2]^+2^ 478.2389 *m*/*z*) vs “ **I** R **H** PYPSY” (AmelX;[M+2]^+2^ 516.7676 *m*/*z*); and (VI) “M­(ox) **L** R **Y** PYP” (AmelY;[M+2]^+2^ 478.2389 *m*/*z*) vs “ **S** M­(ox) **I** R **H** PYP” (AmelX;[M+2]^+2^ 508.7527 *m*/*z*). Modern male sheep: filled green square; modern female sheep: empty green square; modern male goat: filled blue triangle; and modern male goat: empty blue triangle. Neolithic sample from Abu Gosh site males: filled orange dot; Abu Gosh females: empty orange dot. The red line represents the linear regression fit, with *R* ^2^ between 0.948 and 0.997 showing an extremely strong correlation between AmelX and AmelY peptide measurements in modern and ancient samples.

**Figure 3:**
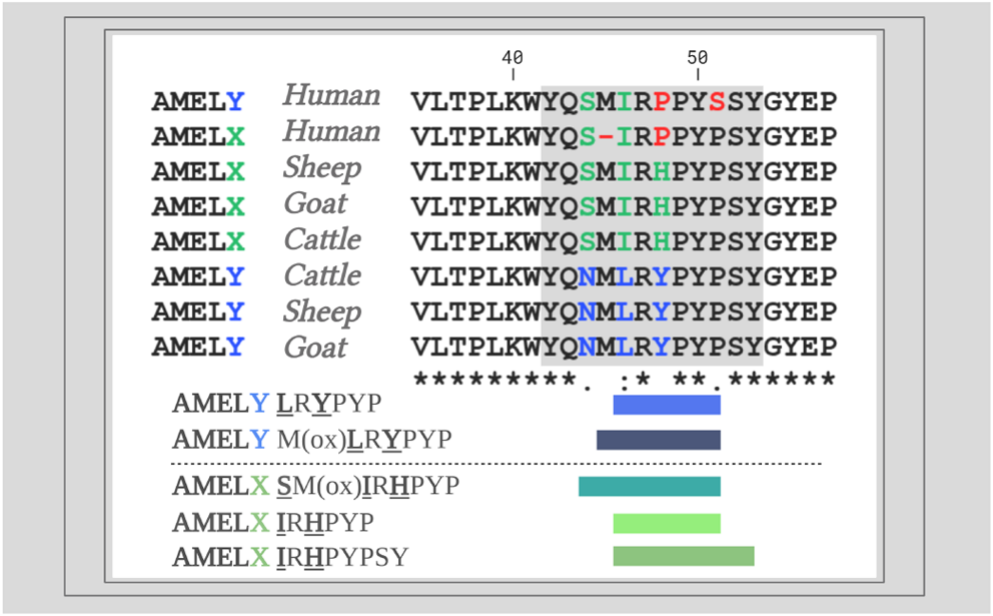
Partial sequence (from positions 35–57) alignment of amelogenin dimorphic proteins from human, cattle, and both caprine sequences (Friesian breed: sheep; Saanen breed: goat). The unique peptide sequence area used in this research is shown in blue (AmelY) and green (AmelX). AmelY unique sequences: “ **L** R **Y** PYP” (AmelY;[M+2]^+2^ 404.7212 *m*/*z*) and “M­(ox) **L** R **Y** PYP” (AmelY;[M+2]^+2^ 478.2389 *m*/*z*); AmelX unique sequences: “ **S** M­(ox) **I** R **H** PYP” (AmelX;[M+2]^+2^ 508.7527 *m*/*z*), “ **I** R **H** PYP” (AmelX;[M+2]^+2^ 391.7191 *m*/*z*), and “ **I** R **H** PYPSY” (AmelX;[M+2]^+2^ 516.7676 *m*/*z*).

#### Selecting Unique Species-Specific Enam Sequences from Modern Samples

An initial MS2 search (Byonic software) was performed to identify unique Enam peptide spectrum matches (PSMs) that distinguish between livestock animal species. Then, we focused on single amino acid variations (SAAVs) between domestic sheep and goat Enam sequence alignments. Sheep Enam at position 187 is occupied by lysine (Lys187) and, at position 212, phenylalanine (Phe212). In contrast, in the goat, the same position in the Enam sequence is occupied by arginine (Arg187) and tyrosine (Try212). We built a list of species-specific target sequences that include at least one of the above unique SAAVs, based on MS2 discovery results. The analysis of the modern samples showed a strong distinction between species (Figure 4). From the extracted data, we were able to select three unique native peptide sequences for modern sheep: IPH **K** IPPGFGRPPG (Enam;[M+3]^+3^ 490.6174 *m*/*z*), **K** IPPGFGRPPG (Enam;[M + 2]^+2^ 561.8246 *m*/*z*) these last including Lys187 and PFFGYFG **F** H (Enam;[M+2]^+2^ 559.7584 *m*/*z*), which incorporates Phe212 as described earlier. For goat determination, we chose two target sequences IPH **R** IPPGFGRPPG (Enam;[M + 3]^+3^ 499.8528 *m*/*z*) and **R** IPPGFGRPPG (Enam;[M+2]^+2^ 575.8276 *m*/*z*). Both sequences incorporate SAAV Arg187. A BLAST search for the unique sequences of Enam (sheep and goat) found that they exhibit homology with Enam proteins from other species such as *Bos indicus* (South Asia, Zebu), *Bos mutus* (Himalaya’s, wild yak), *Bison bison bison* (USA, plains bison) *Erinaceus europaeus* (Western European, hedgehog), *Phascolarctos cinereus* (Australia, Koala), *Odocoileus virginianus texanus* (USA, Texas white-tailed deer), and *Muntiacus reevesi* (China, Reeves’ muntjac) (sequence reviewed by Uniprot March 2025). These species are all nonlocal in the Levant, and/or species that differ significantly in their morphology and biometry from caprines. To confirm the uniqueness of our species identification results in caprines, we included all Enam sequences of animals showing homology, as previously described, in a Byonic search (as outlined in the Materials and Methods section). PSMs identified by Byonic software were manually inspected to verify the peptide assignments. Byonic search results with the extended database yielded correct species identifications, showing high-quality PSMs corresponding to unique species-specific SAAVs, thus confirming the reliability of the associated peptide identifications. This shows the potential of the Enam sequence to differentiate between closely related morphological species such as sheep and goat (caprines).

**Figure 4:**
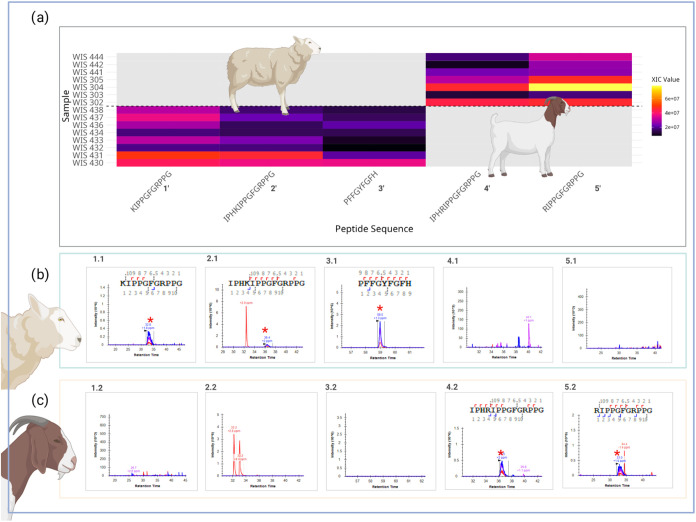
(a) Heatmap showing the abundance (XIC values) of five ENAM peptides in samples of modern sheep (below dashed line) and goat (above dashed line) samples. Color intensity represents XIC (extraction ion chromatogram) values, with bright yellow indicating higher abundance, darker purple indicating lower abundance, and gray indicating undetected peptides, e.g., of the isotopic envelope precursor obtained for (b) sample WIS 430, a modern sheep and (c) sample WIS 305, a modern goat. The peptides (1.1) **K** IPPGFGRPPG (Enam;[M+2]^+2^ 561.8246 *m*/*z*), (1.2) IPH **K** IPPGFGRPPG (Enam;[M+3]^+3^ 490.6174 *m*/*z*), and (1.3) PFFGYFG **F** H (Enam;[M+2]^+2^ 559.7584 *m*/*z*) only exhibit a signal for sheep samples, while (4.2) IPH **R** IPPGFGRPPG (Enam;[M+3]^+3^ 499.8528 *m*/*z*) and (5.2) **R** IPPGFGRPPG (Enam;[M+2]^+2^ 575.8276 *m*/*z*) were detected in goat samples, demonstrating species-specific peptide markers. XIC values are displayed on a continuous scale.

We then extracted ion chromatogram (XIC) intensities from the MS1 data (label-free quantification, LFQ) using Skyline software to confirm the presence of the unique caprine peptide sequences on the DDA extracted chromatograms. The modern sheep and goat samples displayed XIC intensities that were consistent with their distinct Enam peptides; modern sheep show XIC intensities between 7.98 × 10^6^ and 4.92 × 10^7^, while unique goat Enam peptide XIC intensities were between 9.44 × 10^6^ and 7.81 × 10^7^ (see Figure 4 for more details). Note, intensities below 2.5 × 10^5^ were not considered for further analysis and labeled as below the detection threshold. In Figure 5, we present the partial sequence of sheep and goat Enam, along with the alignment of unique, species-specific endogenous peptides selected in this study.

**Figure 5:**
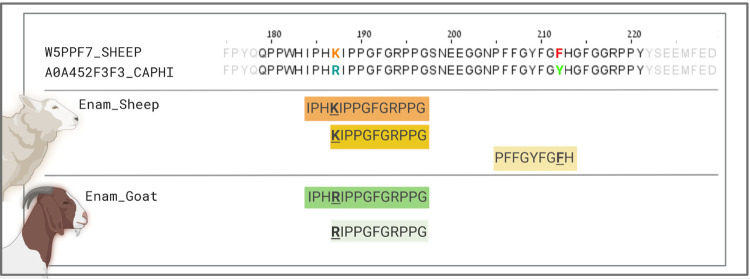
Alignment of partial sequences (from positions 180 to 220) of domestic sheep, *O. aries,* and goat, *C. hircus* Enam sequences (above). In the sheep Enam sequence, position 187 is occupied by lysine (K) and position 212 is occupied by phenylalanine (F). In contrast, the goat Enam sequence shows arginine (R) and tyrosine (Y) at these respective positions. Alignment of Enam unique species-specific native peptides used in this research are shown: domestic sheep: IPH **K** IPPGFGRPPG (Enam;[M+3]^+3^ 490.6174 *m*/*z*); **K** IPPGFGRPPG (Enam;[M+2]^+2^ 561.8246 *m*/*z*); PFFGYFG **F** H (Enam;[M+2]^+2^ 559.7584 *m*/*z*) (center) and goat: IPH **R** IPPGFGRPPG (Enam;[M+3]^+3^ 499.8528 *m*/*z*); **R** IPPGFGRPPG (Enam;[M+2]^+2^ 575.8276 *m*/*z*) (below).

#### Archeological Sample MS Discovery Results

We explored the MS1 chromatogram results for the sexually dimorphic native and species-specific peptides outlined above in ten enamel samples from the Neolithic archeological site of Abu Gosh. All specimens had previously been identified as *Capra* based on morphological criteria;^55^ however, sex had not been determined (for sample details see Table 2). Here, determination of sex was based on DDA identification results of AmelY unique peptides and the presence of at least one unique AmelX sequence. Sex determination was possible in all ten ancient samples (see Figure 2, orange icons). Our results show that four of the ten individuals were males and six females, with XIC intensities for AmelY unique peptides, up to 1.04 × 10^8^ and not more than 2.85 × 10^8^. It is important to note the significant, positive correlation between modern and ancient samples exhibiting unique AmelY peptides (*R* ^2^ = 0.948 and 0.997; Figure 2, plots I–VI).

As illustrated in Figure 1, the unique native sequences of AmelX display an XIC intensity ranging from 7.80 × 10^7^ to 1.91 × 10^9^ (see Abu Gosh archeological samples c.1 and c.2). As an example of sex determination in ancient caprine enamel, we present the results of two ancient caprine enamel chromatograms: sample WIS 424 (Figure 1c.1) that shows the presence of AmelY unique native sequences (chromatograms I and II) alongside the unique native peptides of AmelX (chromatograms III–V) reported as male; and sample WIS 426 (Figure 1c.2) lacking all unique AmelY native peptides (chromatograms I and II) but displaying all three unique AmelX native peptide sequences (chromatograms III–V), thus sexed as female. The above five distinct sequences of five Amel dimorphic peptides and Enam were chosen for the development of the target parallel reaction monitoring (PRM) assay. This decision was based on their enduring stability, evidenced by their consistent presence across both modern and ancient enamel specimens. Deamidation levels of Gln and Asn in the archeological specimens were around 67 and 77% (respectively) compared to the modern caprine samples with levels of 17 and 26%, respectively. This is more than a 40% difference in deamidation occupancy, validating the ancient origin of our samples and the absence of modern contamination between samples. In addition, this result addresses any possible concerns we may have had concerning acid-induced deamidation from sample preparation, since modern samples exhibit low deamidation percentages below 30%.

Finally, the DDA identification results demonstrate the sole presence of species-specific unique goat peptides (see Figure 6 by LFQ [Skyline, software]), which further corroborates the zooarcheological^55^ and previous mitochondrial aDNA^54^ findings for the Abu Gosh assemblage. The ancient samples exhibit unique goat Enam peptide XIC intensities of between 1.39 and 3.70 × 10^8^. Unique native peptides of sheep Enam were absent in all archeological samples from Abu Gosh (see Figure 6a). Furthermore, we included the isotopic envelope precursor of the peptide sequences found in archeological sample WIS 427, incorporating the parallel chromatogram where the unique native peptides of sheep Enam were expected (see Figure 6b, chromatograms 1.1, 2.1, and 3.1), confirming their absence and the presence only of unique goat peptides (see Figure 6c, chromatograms 4.1 and 5.1). Sheep remains from Neolithic archeological sites of similar antiquity were not available for study as this species is uncommon or absent in the region until the mid-seventh millennium BC.^67,68^ Thus, we could not confirm our unique Enam peptide signal on Neolithic sheep.

**Figure 6:**
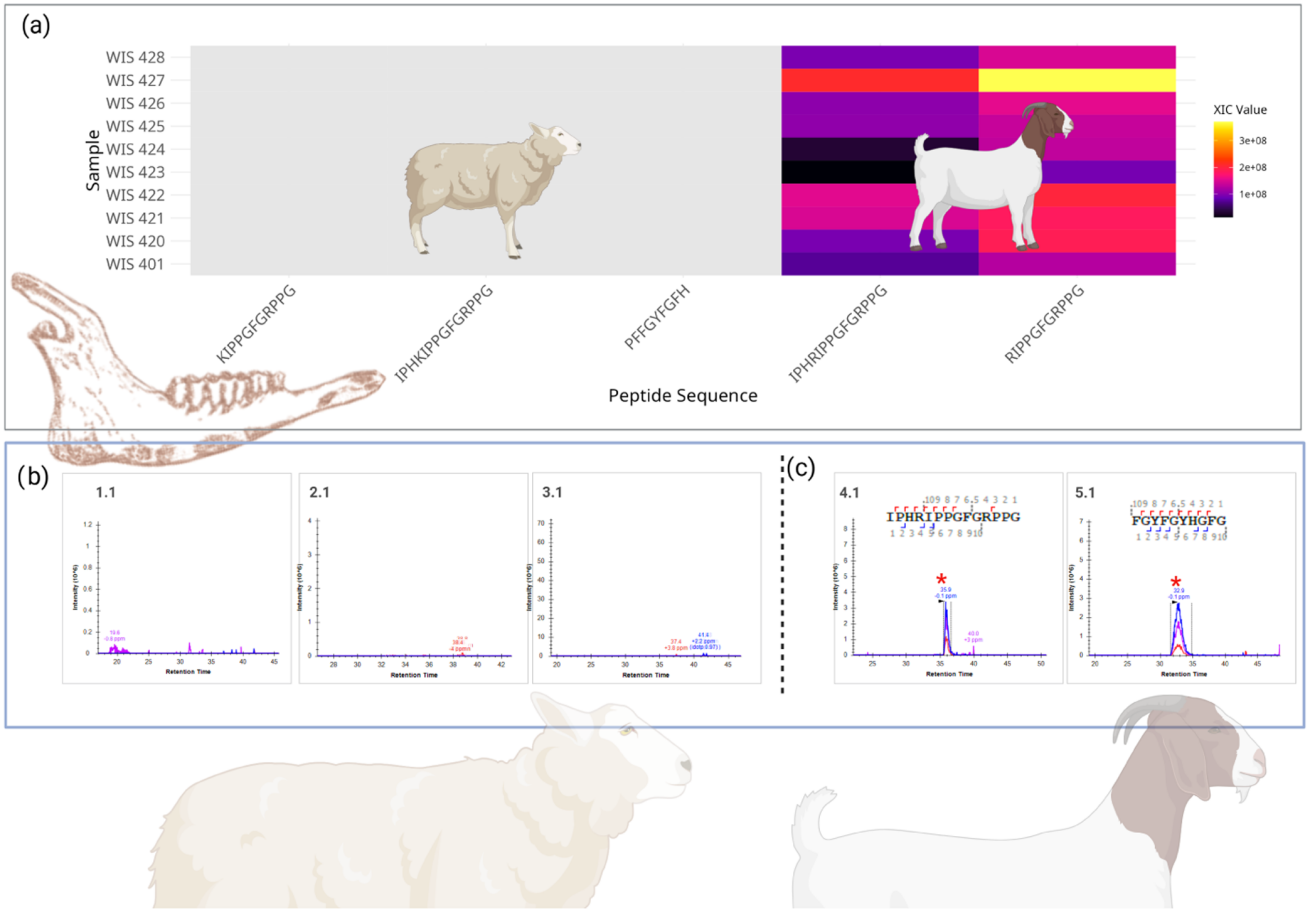
(a) Heatmap showing the abundance (XIC values) of five ENAM peptides in archeological samples from the site of Abu Gosh. Color intensity represents XIC (extraction ion chromatogram) values, with bright yellow indicating higher abundance and darker purple indicating lower abundance. Gray titles indicate undetected peptides. For the ancient sample of Abu Gosh (WIS 427), sheep unique peptide, (b) isotopic envelope precursors were absent in the sample studied (1.1) **K** IPPGFGRPPG (Enam;[M + 2]^+2^ 561.8246 *m*/*z*), (2.1) IPH **K** IPPGFGRPPG (Enam;[M + 3]^+3^ 490.6174 *m*/*z*), and (3.1) PFFGYFG **F** H (Enam;[M + 2]^+2^ 559.7584 *m*/*z*). However, unique goat peptide sequences (c) (4.2) IPH **R** IPPGFGRPPG (Enam;[M + 3]^+3^ 499.8528 *m*/*z*) and (5.2) **R** IPPGFGRPPG (Enam;[M + 2]^+2^ 575.8276 *m*/*z*) were detected in the ancient sample, demonstrating species-specific peptide markers. XIC values are displayed on a continuous scale.

### Application of Parallel Reaction Monitoring (PRM) Assay for Caprine Speciation and Sex

#### Selected Peptide Results for Sex Determination in Modern Samples

Our target approach gave results similar to those of the previous DDA analysis for sex determination. A total of five target peptide sequences were selected: two unique native dimorphic peptides of AmelY (“ **L** R **Y** PYP” and “M­(ox) **L** R **Y** PYP”) and three native dimorphic peptides of AmelX (“ **S** M­(ox) **I** R **H** PYP”, “ **I** R **H** PYPSY”, and “ **I** R **H** PYP”). Similar to our DDA results, AmelY unique native peptide sequences were detected only in male goat samples with XIC intensities reaching 3.19 × 10^6^. In modern samples, the unique AmelX peptides show XIC intensities of between 3.34 × 10^5^ and 3.88 × 10^7^.

#### Selected Peptide Results for Species Determination in Modern Samples

Based on the previously selected peptides from the DDA runs, we developed a targeted assay for caprine species determination only. Targeted analysis of these unique Enam sequences in modern caprine samples gave results identical with those from the previous DDA analysis. Our PRM results of unique Enam peptides for modern sheep and goats exhibited XIC intensities characteristic of their species-specific target sequences. Modern sheep had XIC intensities between 4.92 × 10^6^ and 1.02 × 10^8^ and modern goats intensities between 7.56 × 10^4^ and 4.92 × 10^7^. Using the PRM target proteomics assay, at least one unique Enam native peptide per sample was found to be present for species determination in modern samples.

#### Sex Determination and Further Species Confirmation in Archeological Samples

The PRM of all ten enamel samples from the Abu Gosh archeological site confirmed the previous results obtained by the DDA method for sex determination as well as confirming Enam utility for species determination. Amel peptides in these ancient samples show intensities at least one magnitude higher than those of modern caprine samples. XIC intensities of AmelX peptides in the archeological samples were between 3.32 × 10^7^ and 3.72 × 10^8^ and the intensities of unique AmelY peptides were up to 7.54 × 10^7^ (Figure 7). We also established the male sex of four of ten of the ancient Neolithic samples. In addition, a significant positive correlation was identified between modern and ancient samples that exhibited the presence of unique AmelY peptides (*R* ^2^ = 0.987). Finally, we confirmed species identification based on zooarcheology and mtDNA analyses^54,55^ that determined that goats (*Capra*) are the only ancient caprines represented at this Neolithic site. This was established due to the presence of both unique peptides (XIC intensities between 1.25 and 7.96 × 10^7^) and the absence of unique sheep peptide sequences. Figure 8 presents the XIC intensity signals for the unique species-specific peptides for all samples (modern and ancient), illustrating the specificity of the relevant peptides. It also shows the species-specific MS2 chromatogram fragmentation results and confirms the sequences obtained using PRM.

**Figure 7:**
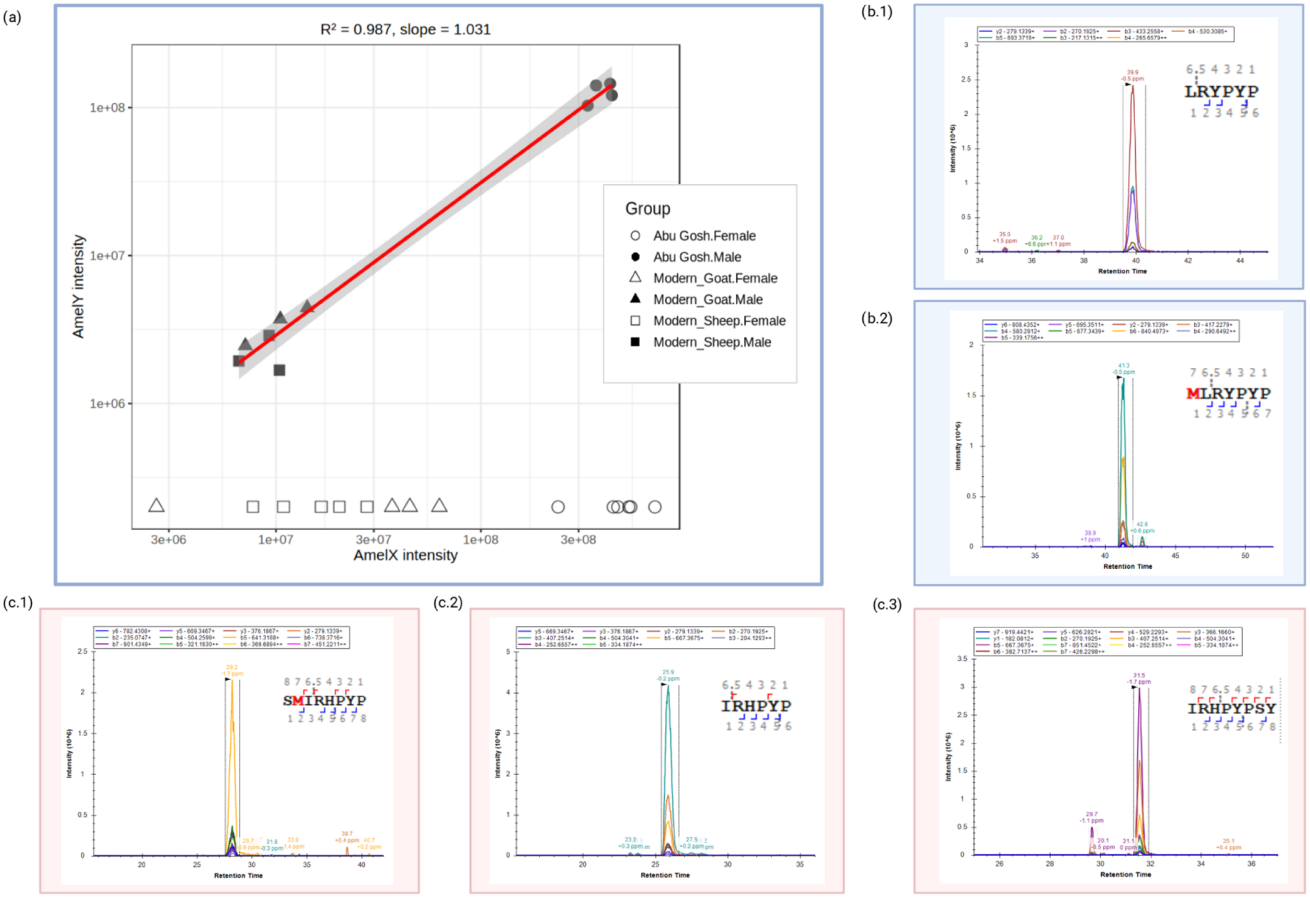
(a) Scatter plot illustrating the relationship between AmelX and AmelY intensities for all modern and archeological (Abu Gosh) samples. The solid red line represents the linear regression fit for all samples with AmelY signals, indicating a strong positive correlation (*R* = 0.987) irrespective of group affiliation. MS2 trace chromatograms obtained using Skyline software on target Amel peptide sequences: (b.1 and b.2) unique caprine AmelY peptides “ **L** R **Y** PYP” and “M­(ox) **L** R **Y** PYP”; and AmelX unique peptides (c.1) “ **S** M­(ox) **I** R **H** PYP”, (c.2) “ **I** R **H** PYP”, and (c.3) “ **I** R **H** PYPSY”.

**Figure 8:**
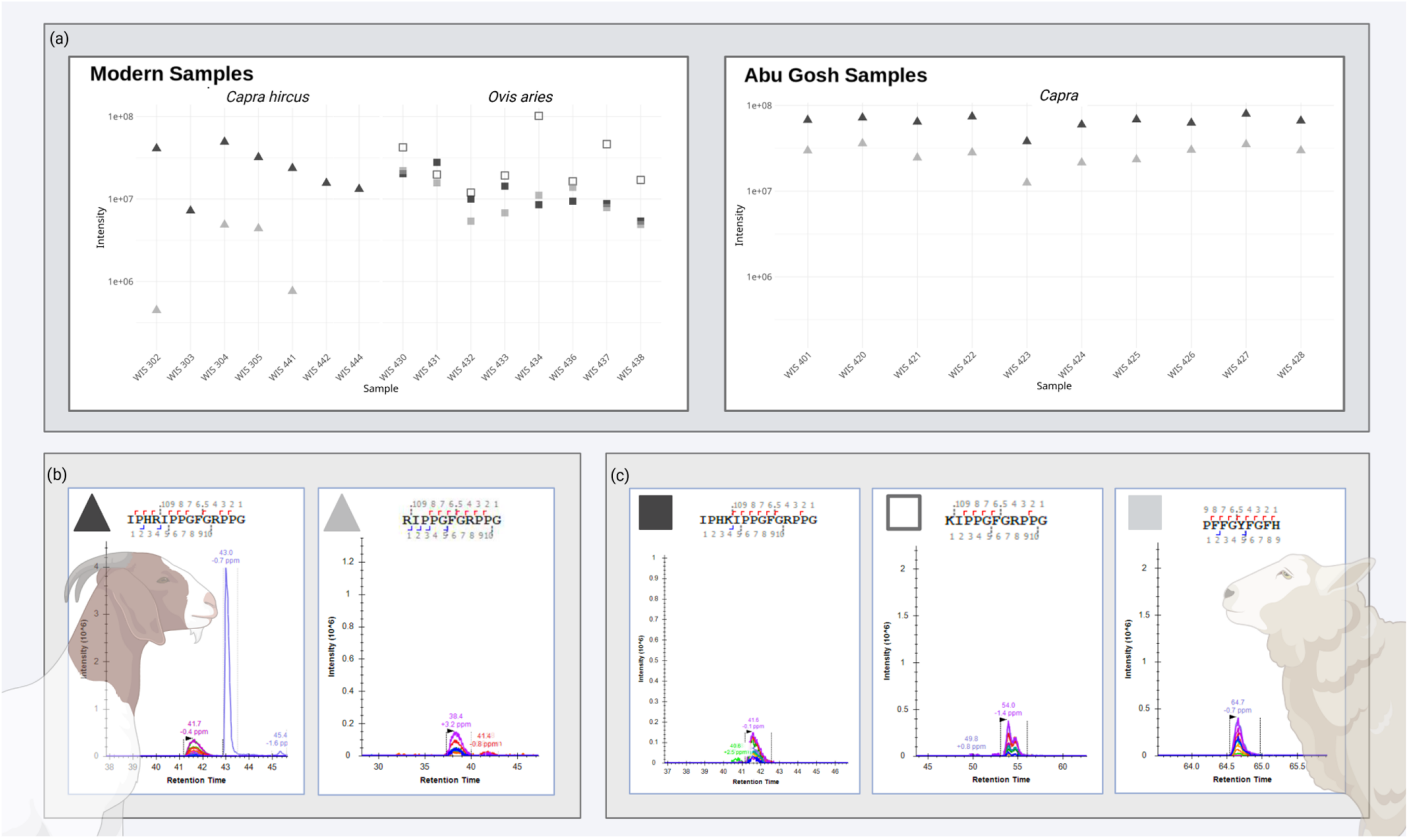
(a) Plot displays the intensity values of Enam unique species-specific peptides for modern domestic sheep and goat samples (left) and archeological samples from the Neolithic site of Abu Gosh (right). Squares indicate species-specific *Ovis* peptides, and triangles denote species-specific *Capra* peptides. Missing values are noted as below the detection threshold (XIC below 2 × 10^5^). Species-specific Enam peptides MS2 chromatogram and its fragmentation are as follows: (b) *C. hircus* IPH **R** IPPGFGRPPG (Enam;[M + 3]^+3^ 499.8528 *m*/*z*); **R** IPPGFGRPPG (Enam;[M + 2]^+2^ 575.8276 *m*/*z*). (c) *O. aries*: IPH **K** IPPGFGRPPG (Enam;[M + 3]^+3^ 490.6174 *m*/*z*); **K** IPPGFGRPPG (Enam;[M + 2]^+2^ 561.8246 *m*/*z*); PFFGYFG **F** H (Enam;[M + 2]^+2^ 559.7584 *m*/*z*).

## Discussion

This study advances the application of enamel proteomics by establishing a targeted approach for sex determination and the confirmation of species identification in modern and ancient caprines. Using single amino acid variations (SAAVs) in amelogenin (Amel) and enamelin (Enam) peptides, we identified reliable target peptide sequences in both modern and archeological samples. For sex determination, our targeted analysis confirmed the reliability of Amel peptides, to identify male-specific AmelY peptides “ **L** R **Y** PYP” and “M­(ox) **L** R **Y** PYP”, in both modern and ancient samples. For the archeological samples, we demonstrated a predominance of females (six of the ten samples). Our findings align with previous research demonstrating the potential of Amels as a sex-specific biomarker in modern and ancient mammals.^4,43,53^ Insights into the sex ratios of archeological caprine remains can provide demographic data enabling an exploration of domestic herd management strategies, i.e., for meat production versus milk or wool/hair production or a combination of these, as each strategy has a characteristic sex composition^69–71^ . Additionally, sex ratios might illustrate selection in hunting practices, such as targeting male animals for their horns or hunting in specific seasons.^72–75^

The preservation of AmelY peptides in the archeological samples suggests that the mineral matrix of the enamel may protect these molecular markers, preserving sex-specific sequences even under adverse diagenetic conditions.^19^ Indeed, the clear XIC signals from AmelY peptides indicate that specific amelogenin sequences may withstand degradation better than those of other peptides. This supports prior studies suggesting that enamel provides a stable environment for protein preservation over extensive timeframes.^2,32^ Nevertheless, our study reveals that post translational modifications (PTMs) and fragmentation remain challenging, particularly in samples with a high degree of diagenesis. It is important to note that archeological samples show higher peptide intensity due to diagenesis, which has increased the peptide fragmentation from long peptides to short ones.^1,3,28^ Further research could expand on these findings by assessing diagnostic impacts under controlled conditions.

For species confirmation, Enam peptides successfully distinguished sheep (*O. aries*) from goats (*C. hircus*) in our modern samples, and are consistent with the sole presence of goats in the early Neolithic site of Abu Gosh as determined by ancient DNA^54^ as well as zooarcheology.^55,56^ Our results build on those of Bray et al., who showed that enamelin peptides are well-preserved and can differentiate genera such as *Bos* and *Bison* in fossils over 120,000 years old. While their work demonstrated genus-level resolution, our study refines this approach to distinguish between *O. aries* and *C. hircus* at the species level, highlighting enamelin’s value for both deep-time taxonomy and domestic animal identification in archeological contexts. Notably, our study is one of the first applications of amino acid sequences recovered from enamelin (Enam) for caprine species identification.

Although ZooMS has become a cornerstone of archeological science, offering a fast and accessible way to identify taxa based on collagen peptide fingerprints,^6,8,11^ its utility is reliant on the survival of collagen—a protein that degrades readily in warm, acidic, or humid burial environments.^19,76^ In contrast, enamel provides a much more stable matrix. Composed of approximately 95% hydroxyapatite, enamel traps a small set of proteins like amelogenin and enamelin during tooth development and protects them from microbial and chemical degradation.^25,32^ As demonstrated here, these proteins can persist for thousands of years and contain sequence-level differences sufficient for both species and sex identification. Thus, our findings align with prior research underscoring the potential of enamel for molecular taxonomic identification and confirms enamel proteomics as an alternative to ZooMS,^1,3,19,56^ particularly in degraded samples where collagen is poorly preserved or absent.

Our approach takes advantage of this preservation through a two-step strategy: discovery by label-free quantification (LFQ), followed by parallel reaction monitoring (PRM) for results confirmation. Crucially, this method analyzes native peptides directly, without enzymatic digestion, reducing the risk of introducing biases during sample preparation. LFQ enables quantitative comparisons and peptide discovery, while PRM offers high-precision validation of specific peptide sequences. This strategy allowed us to detect species variants in enamelin and sex-linked peptides in amelogenin across all archeological samples, something that ZooMS cannot achieve.

However, our method is currently tailored for caprines, having been designed by using modern sheep and goat enamel and reference sequences. While our targeted enamelin peptides proved reliable for distinguishing sheep and goats, it is important to acknowledge a key limitation of this approach: the potential for false positives due to sequence similarity in Enam proteins across other mammalian taxa. A BLAST search of our unique Enam peptide sequences revealed some partial sequence homology with species outside the caprine clade, but most of these were morphologically or metrically different and/or geographically distant to our targeted taxa, i.e., did not inhabit the Levant during the Neolithic. Additionally, we cross-validated our results with morphological and aDNA data, providing multiple lines of evidence for taxonomic assignment. However, in other contexts, especially where faunal assemblages include a broader range of wild bovids or cervids, this limitation could pose a risk.

To address this issue, future work should prioritize the expansion of enamel protein reference databases, particularly for wild species. Comparative proteogenomic analyses can help identify species-specific SAAVs. Furthermore, using a multimarker approach that targets peptides across several enamel proteins (e.g., ameloblastin, tuftelin) would reduce reliance on SAAVs and lower the risk of misclassification. Finally, integrating enamel proteomics with complementary methods such as ZooMS, aDNA, or morphometric analysis of animal remains may be essential when working with taxonomically diverse or poorly preserved assemblages. In addition, incorporating comparative analyses of different taxa and geographic areas will be crucial for developing this approach into a broad-based application for archeological science.

In summary, while ZooMS remains an invaluable technique for collagen-rich materials, enamel proteomics offers a powerful and complementary alternative for degraded samples. Our combined LFQ+PRM workflow enables fine-scale identification using one of the most stable biomaterials available in the archeological record. As shown here, it can recover valuable taxonomic and biological information from specimens that may otherwise be inaccessible.

## Conclusions

This study represents a significant advance in enamel proteomics, introducing a robust methodology for sex determination in modern and ancient caprines and offering a complementary method of species identification. By developing a targeted parallel reaction monitoring (PRM) assay focusing on sex-specific Amel peptides and the potential of Enam peptide species-specific unique peptides, we provide an accurate and reliable tool for sex determination based on the presence of AmelY peptides (sheep and goat) as well as the ability to differentiate sheep from goats. Validating these markers in archeological samples from the eighth millennium BC site of Abu Gosh further emphasizes this method’s relevance to archeology and paleontology and shows how enamel proteomics can complement traditional zooarcheological and aDNA methods. Thus, by establishing a framework for targeted enamel proteomics and including enamelin analyses in our study for the first time, we have expanded the molecular toolkit available for ancient biomolecular research. Our findings substantiate enamel as a durable, taxonomically informative substrate, demonstrating that enamel proteins retain significant molecular detail for species and sex determination even in degraded samples.

## Supplementary Material





## Data Availability

The mass spectrometry proteomics data have been deposited in the ProteomeXchange Consortium via the PRIDE^77^ partner repository with following data set identifiers: (1) PXD058275, 10.6019/PXD058275 and (2) PXD058279, 10.6019/PXD058279. **ProteomeXchange via the PRIDE database**: The data was divided into two projects; see the details below. **(a) Project Name:** Part I: Proteomics Target Approach for Taxonomic and Sex Identification in Modern and Archeological Enamel Caprines. **Project accession:** PXD058275; **Project DOI:** 10.6019/PXD058275. (**b) Project Name:** Part II: Proteomics Target Approach for Taxonomic and Sex Identification in Modern and Archeological Enamel Caprines. **Project accession:** PXD058279; **Project DOI:** 10.6019/PXD058279

## Notes

Experimental workflow and additional results comprising images of modern reference specimens including female and male sheep and goat crania (SI Figures 1–3); enamel sampling from female goats (SI Figure 4); archeological caprine specimens from Abu Gosh (SI Figures 5–7); condition of teeth before and after mechanical cleaning (SI Figure 8); cleaned enamel samples from modern sheep and goats (SI Figures 9–11); and the enamel extraction process (SI Figure 12); and bioinformatic alignments of AmelX and AmelY proteins across species and sheep breeds (SI Figures 13 and 14) (PDF)
Predicted CDs (*O. aires* and *C. hircus*) at “Supplemental file X full AMEL sequences” (TXT)

## References

[1] Cappellini E., Welker F., Pandolfi L. (2019). Early Pleistocene enamel
proteome from Dmanisi resolves *Stephanorhinus* phylogeny. Nature.

[2] Cleland T. P., Schroeter E. R., Colleary C. (2021). Diagenetiforms: a new
term to explain
protein changes as a result of diagenesis in paleoproteomics. J. Proteomics.

[3] Taurozzi A. J., Rüther P. L., Patramanis I. (2024). Deep-time phylogenetic
inference by paleoproteomic analysis of dental enamel. Nat. Protoc..

[4] Berezina N., Ziganshin R., Kolobova K. (2024). Bison sex matters: the
potential of proteomic tooth enamel analysis for determination of
ancient human subsistence strategies. Archaeol.
Anthropol. Sci..

[5] Buckley M., Pigière F., Chowdhury M. P., Kitchener A., Smyth J. (2024). Proteomic sexing of
archaeological cattle remains at Neolithic Kilshane. J. Archaeol. Sci..

[6] Buckley M., Collins M., Thomas-Oates J., Wilson J. C. (2009). Species identification
by analysis of bone collagen using matrix-assisted laser desorption/ionisation
time-of-flight mass spectrometry. Rapid Commun.
Mass Spectrom..

[7] Collins, M. ZooMS: the collagen barcode and fingerprints Spectrosc. Eur. 2010; Vol. 22 6.

[8] Richter K. K., Wilson J., Jones A. K. (2011). Fish’n chips:
ZooMS peptide mass fingerprinting in a 96 well plate format to identify
fish bone fragments. J. Archaeol. Sci..

[9] Buckley M., Fraser S., Herman J. (2014). Species identification
of archaeological marine mammals using collagen fingerprinting. J. Archaeol. Sci..

[10] Welker F., Soressi M., Rendu W., Hublin J.-J., Collins M. (2015). Using ZooMS
to identify fragmentary bone from the late Middle/Early Upper Palaeolithic
sequence of Les Cottés, France. J. Archaeol.
Sci..

[11] Welker F., Collins M. J., Thomas J. A. (2015). Ancient proteins resolve
the evolutionary history of Darwin’s South American ungulates. Nature.

[12] Desmond A., Barton N., Bouzouggar A. (2018). ZooMS identification
of bone tools from the North African Later Stone Age. J. Archaeol. Sci..

[13] Buckley, M. Zooarchaeology by Mass Spectrometry (Zooms) Collagen Fingerprinting for the Species Identification of Archaeological Bone Fragments. In Zooarchaeology in Practice: Case Studies in Methodology and Interpretation in Archaeofaunal Analysis; Giovas, C. M. ; LeFebvre, M. J. , Eds.; Springer International Publishing: Cham, 2018; pp 227–247.

[14] Brandt L. Ø., Haase K., Collins M. J. (2018). Species
identification using ZooMS,
with reference to the exploitation of animal resources in the medieval
town of Odense. Dan. J. Archaeol..

[15] Pilaar
Birch S. E., Scheu A., Buckley M., Çakırlar C. (2019). Combined osteomorphological,
isotopic, aDNA, and ZooMS analyses of sheep and goat remains from
Neolithic Ulucak, Turkey. Archaeol. Anthropol.
Sci..

[16] Atavliyeva S. S., Tarlykov P. (2021). Paleoproteomics studies of ancient Caprinae: a review. Exp. Biol..

[17] Culley C., Janzen A., Brown S. (2021). Iron Age hunting and
herding in coastal eastern Africa: ZooMS identification of domesticates
and wild bovids at Panga ya Saidi, Kenya. J.
Archaeol. Sci..

[18] Coutu A. N., Taurozzi A. J., Mackie M. (2021). Palaeoproteomics
confirm
earliest domesticated sheep in southern Africa ca. 2000 bp. Sci. Rep..

[19] Hendy J., Welker F., Demarchi B. (2018). A guide to ancient protein
studies. Nat. Ecol. Evol..

[20] Chen, H. ; Liu, Y. Advanced Ceramics for Dentistry; Elsevier, 2014; pp 5–21.

[21] Sire J.-Y., Davit-Béal T., Delgado S., Gu X. (2007). The origin and evolution
of enamel mineralization genes. Cells Tissues
Organs.

[22] Sakae T., Hirai G. (1982). Calcification and crystallization
in bovine enamel. J. Dental Res..

[23] Smith C. E. (1998). Cellular
and chemical events during enamel maturation. Crit. Rev. Oral Biol. Med..

[24] Suga, S. Comparative histology of the progressive mineralization pattern of developing enamel. In Mechanisms of Tooth Enamel Formation; Suga, S. , Ed.; Quintessence Publishing: Tokyo, 1983; pp 167–203.

[25] Nanci, A. Ten Cate’s Oral Histology: Development, Structure and Function; Mosby: St. Louis, 2007.

[26] Moradian-Oldak J. (2012). Protein-mediated
enamel mineralization. Front. Biosci. A:.

[27] Nogueira F. C., Neves L. X., Pessoa-Lima C. (2021). Ancient enamel peptides
recovered from the South American Pleistocene species *Notiomastodon
platensis* and *Myocastor* cf. *coypus*. J. Proteomics.

[28] Welker F., Ramos-Madrigal J., Kuhlwilm M. (2019). Enamel proteome shows
that *Gigantopithecus* was an early diverging pongine. Nature.

[29] He L. H., Swain M. V. (2008). Understanding the mechanical behaviour
of human enamel
from its structural and compositional characteristics. J. Mech. Behav. Biomed. Mater..

[30] Kohn M. J., Schoeninger M. J., Barker W. W. (1999). Altered states:
effects of diagenesis
on fossil tooth chemistry. Geochim. Cosmochim.
Acta.

[31] Thomas D. B., McGoverin C. M., Fordyce R. E., Frew R. D., Gordon K. C. (2011). Raman spectroscopy
of fossil bioapatite proxy for diagenetic alteration of the oxygen
isotope composition. Palaeogeogr. Palaeoclimatol.
Palaeoecol..

[32] Demarchi B., Hall S., Roncal-Herrero T. (2016). Protein sequences bound
to mineral surfaces persist into deep time. eLife.

[33] Pajares G., Álvarez I., Fernández I. (2007). A sexing protocol for
wild ruminants based on PCR amplification of amelogenin genes AMELX
and AMELY. Arch. Animal Breed..

[34] Fontanesi L., Scotti E., Russo V. (2008). Differences
of the porcine amelogenin
X and Y chromosome genes (AMELX and AMELY) and their application for
sex determination in pigs. Mol. Reprod. Dev..

[35] Brinkman T. J., Hundertmark K. J. (2009). Sex identification
of northern ungulates using low
quality and quantity DNA. Conserv. Genet..

[36] Fiore A. D. (2006). A rapid
genetic method for sex assignment in non-human primates. Conserv. Genet..

[37] Afonso C., Nociarova D., Santos C. (2019). Sex selection
in late
Iberian infant burials: integrating evidence from morphological and
genetic data. Am. J. Hum. Biol..

[38] Ennis S., Gallager T. F. (1994). A PCR-based sex-determination assay in cattle based
on the bovine amelogenin locus. Animal Genet..

[39] Pfeiffer I., Brenig B. (2005). X- and Y-chromosome
specific variants of amelogenin
gene allow sex determination in sheep (*Ovis aries*) and European red deer (*Cervus elaphus*
*)*. BMC Genet..

[40] Weikard R., Pitra C., Kühn C. (2006). Amelogenin cross-amplification in
the family Bovidae and its application for sex determination. Mol. Rep. Dev..

[41] Dervishi E. (2008). Reliability of sex determination in ovine embryos using amelogenin
gene (Amels). Theriogenology.

[42] Tsai T. C., Wu S. H., Chen H. L. (2011). Identification of sex-specific
polymorphic sequences in the goat amelogenin gene for embryo sexing. J. Animal Sci..

[43] Stewart N. A., Molina G. F., Issa J. P. M. (2016). The identification of
peptides by nanoLC-MS/MS from human surface tooth enamel following
a simple acid etch extraction. RSC Adv..

[44] Stewart N. A., Gerlach R. F., Gowland R. L., Gron K. J., Montgomery J. (2017). Sex determination
of human remains from peptides in tooth enamel. Proc. Natl. Acad. Sci. U.S.A..

[45] Lugli F., Di Rocco G., Vazzana A. (2019). Enamel peptides reveal
the sex of the Late Antique ’Lovers of Modena’. Sci. Rep..

[46] Froment C., Hourset M., Sáenz-Oyhéréguy N. (2020). Analysis of 5000 year-old human teeth using optimized large-scale
and targeted proteomics approaches for detection of sex-specific peptides. J. Proteomics.

[47] Wasinger V. C., Curnoe D., Bustamante S. (2019). Analysis of the preserved
amino acid bias in peptide profiles of Iron Age teeth from a tropical
environment enable sexing of individuals using amelogenin MRM. Proteomics.

[48] Parker G. J., Yip J. M., Eerkens J. W. (2019). Sex estimation using
sexually dimorphic amelogenin protein fragments in human enamel. J. Archaeol. Sci..

[49] Welker F., Ramos-Madrigal J., Gutenbrunner P. (2020). The dental proteome
of *Homo antecessor*. Nature.

[50] Demeter F., Zanolli C., Westaway K. E. (2022). A Middle Pleistocene
Denisovan molar from the Annamite chain of northern Laos. Nat. Commun..

[51] Zazueta, R. F. ; Krueger, F. ; Alba, D. M. Phylogenetic signal in primate tooth enamel proteins and its relevance for paleoproteomics BioRxiv 2024. 10.1093/gbe/evaf007 PMC11878541 39834226

[52] Madupe, P. P. ; Munir, F. ; Dickinson, M. Results from an australopithecus africanus dental enamel fragment confirm the potential of palaeoproteomics for South African Plio-Pleistocene fossil sites S. Afr. J. Sci. 2025; Vol. 121 10.17159/sajs.2025/18571.

[53] Kotli P., Morgenstern D., Bocquentin F. (2024). A label-free quantification
method for assessing sex from modern and ancient bovine tooth enamel. Sci. Rep..

[54] Kahila
Bar-Gal G., Khalaily H., Mader O., Ducos P., Horwitz L. K. (2002). Ancient DNAevidence for the transition from wild to
domestic status in Neolithic goats: a case study from the site of
Abu Gosh, Israel. Ancient Biomol..

[55] Ducos, P. ; Horwitz, L. K. Pre Pottery Neolithic B fauna from the Lechevallier Excavations at Abu-Ghosh. In The Neolithic Site of Abu Gosh. The 1995 Excavations; Khalaily, H. ; Marder, O. , Eds., Reports; Israel Antiquities Authority: Jerusalem, 2003; Vol. 19, pp 103–120.

[56] Bray F., Julien M. A., Delegue L. (2025). Simultaneous taxonomic
and sex identification of *Bos* and *Bison* teeth using low-invasive high-resolution mass spectrometry. J. Proteome Res..

[57] Green D. R., Uno K. T., Miller E. R. (2025). Eighteen
million years
of diverse enamel proteomes from the East African Rift. Nature.

[58] Paterson R. S., Mackie M., Capobianco A. (2025). Phylogenetically informative
proteins from an early miocene rhinocerotid. Nature.

[59] Tsai T.-C. (2011). Identification of sex-specific
polymorphic sequences in the goat
amelogenin gene for embryo sexing. J. Animal
Sci..

[60] Hu J.-C., Zhang C., Yang Y. (2001). Cloning and characterization
of the mouse and human enamelin genes. J. Dental
Res..

[61] Lechevallier, M. ; Arensburg, M. ; Le Brun, B. Abou Gosh et Beisamoun. Deux gisements du VII millénaire avant l’ère Chrétienne en Israël, Mémoires et Travaux du Centre de Recherches Préhistoriques Français de Jérusalem; CNRS: 1978; 340.

[62] Danin, A. Flora and vegetation of Israel and adjacent areas. Vol. Dr. W. Junk Publishers Dordrecht: The Netherlands, 1988; Vol. 30, pp 251–276.

[63] The Neolithic Site of Abu Gosh. In The Neolithic Site of Abu Gosh. The 1995 Excavations; Khalaily, H. ; Marder, O. , Eds.; Israel Antiquities Authority Reports: Israel Antiquities Authority, Jerusalem, 2003; Vol. 9.

[64] Helmer D. (2000). Discrimination
des genres *Ovis* et *Capra* à
l’aide des prémolaires inférieures 3 et 4 et
interprétation des âges d’abattage: l’exemple
de Dikili Tash (Grèce). Anthropozoologica.

[65] Halstead P., Collins P., Isaakidou V. (2002). Sorting the
sheep from the goats:
morphological distinctions between the mandibles and mandibular teeth
of adult *Ovis* and *Capra*. J. Archaeol. Sci..

[66] Edgar R. C. (2004). Muscle:
multiple sequence alignment with high accuracy and high throughput. Nucleic Acids Res..

[67] Martin, L. ; Edwards, Y. Diverse Strategies: Evaluating the appearance and spread of domestic caprines in the Southern Levant. In The Origins and Spread of Domestic Animals in Southwest Asia and Europe; Routledge, 2016; pp 49–82.

[68] Gourichon L., Horwitz L. K. (2021). An inter-regional
comparison of animal domestication
in the northern and southern levant. Food Hist..

[69] Payne S. (1973). Kill-off patterns
in sheep and goats: the mandibles from Aşvan Kale. Anatolian studies.

[70] Redding, R. W., Jr Decision making in subsistence herding of sheep and goats in the Middle East, Ph.D. Thesis; University of Michigan, 1981.

[71] Zeder M. A. (2001). A metrical
analysis of a collection of modern goats (*Capra hircus aegargus* and *C. h. hircus*) from Iran and Iraq: implications
for the study of caprine domestication. J. Archaeol.
Sci..

[72] Chaix, L. ; Desse, J. Les bouquetins de l’observatoire (Monaco) et des Baoussé Roussé (Grimaldi, Italie). première partie: cranium, atlas, epistropheus Bulletin du Musée d’Anthropologie Préhistorique de Monaco Monaco;. Bulletin du Musée d’Anthropologie Préhistorique de Monaco Monaco; OCLC, **1982**, 41–74.

[73] Monchot H. (1999). Mixture analysis
and mammalian sex ratio among Middle Pleistocene mouflon of Arago
cave, France. Quat. Res..

[74] Deakin S., Festa-Bianchet M., Miller J. M., Pelletier F., Coltman D. W. (2022). Ewe are what ewe
wear: bigger horns, better ewes and
the potential consequence of trophy hunting on female fitness in bighorn
sheep. Proc. Royal Soc. B.

[75] Singer F. J., Zeigenfuss L. C. (2002). Influence of trophy hunting and horn size on mating
behavior and survivorship of mountain sheep. J. Mammal..

[76] Weiner, S. Microarchaeology: beyond the visible archaeological record; Cambridge University Press, 2010.

[77] Perez-Riverol Y., Bai J., Bandla C. (2022). The pride database resources in 2022: a hub
for mass spectrometry-based proteomics evidences. Nucleic Acids Res..

