# Supplementary material for: Sex Identification and Species Confirmation in Modern and Archeological Caprine Enamel
Source: J Proteome Res. 2025 Aug 6;24(9):4403–16. doi: 10.1021/acs.jproteome.5c00012 (PMC12418508; doi:10.1021/acs.jproteome.5c00012)
Supplement: Supplementary file 1 [file pr5c00012_si_002.pdf]

Supplementary Information (SI): Sex Identification and Species Confirmation in Modern and Archaeological Caprine Enamel

Paula Kotli[1,*], David Morgenstern[2], Shifra Ben-Dor[3], Liora Kolska Horwitz[4], and Elisabetta Boaretto[1,*]

[1] *Scientific Archaeology and D-REAMS Radiocarbon Dating Laboratory, Weizmann Institute of Science, 760001, Rehovot, Israel*
[2] *Nancy and Stephen Grand Israel National Center for Personalized Medicine G-INCPM, Weizmann Institute of Science, 760001, Rehovot, Israel*
[3] *Bioinformatics Unit, Life Sciences Core Facilities, Weizmann Institute of Science, Rehovot 76100, Israel*
[4] *National Natural History Collections, E. Safra-Givat Ram Campus, The Hebrew University of Jerusalem, 96194 Jerusalem, Israel*
[*] *paula.kotli@weizmann.ac.il; kotli@mail.tau.ac.il; elisabetta.boaretto@weizmann.ac.il*

Table of Contents

SI Figure 1: Six female *Ovis aries* heads as received from Shefa-Amr slaughterhouse 2
SI Figure 2: Three male *Ovis aries* heads as received from Shefa-Amr slaughterhouse 3
SI Figure 3: Three male *Capra hircus* heads as received from Shefa-Amr slaughterhouse 4
SI Figure 4: Female goat samples (WIS302-WIS305) 4
SI Figure 5: Abu Gosh samples (WIS420-422) 5
SI Figure 6: Abu Gosh samples (WIS423-426) 6
SI Figure 7: Abu Gosh samples (WIS427-429) 7
SI Figure 8: Teeth before/after cleaning (24.1.001) 8
SI Figure 9: Extracted sheep incisors (24.1.001-006) 9
SI Figure 10: Extracted sheep incisors (24.2.001-003) 9
SI Figure 11: Extracted goat incisors (24.3.001- 24.3.004 10
SI Figure 12: Enamel extraction process (24.2.002) 11
SI Figure 13: Alignment of AmelX/AmelY (multiple species) 12
SI Figure 14: Alignment of AmelX/AmelY (sheep breeds) 13

1 Animal Crania as Received from Shefa-Amr Slaughterhouse, Israel.

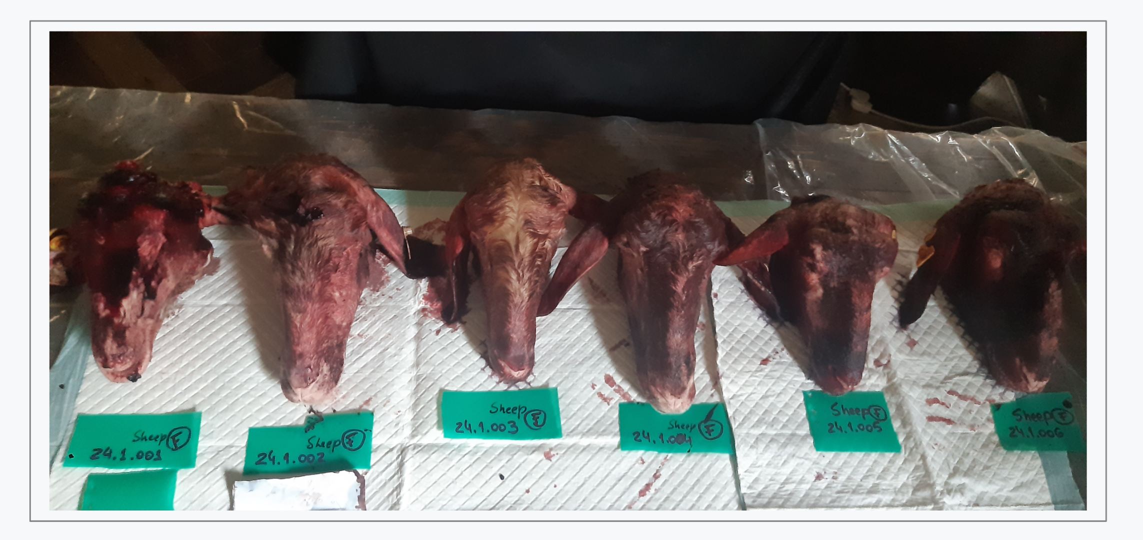

SI Figure 1: Six female *Ovis aries* (sheep) head as received from the Slaughterhouse, Shefa-Amr, Israel.

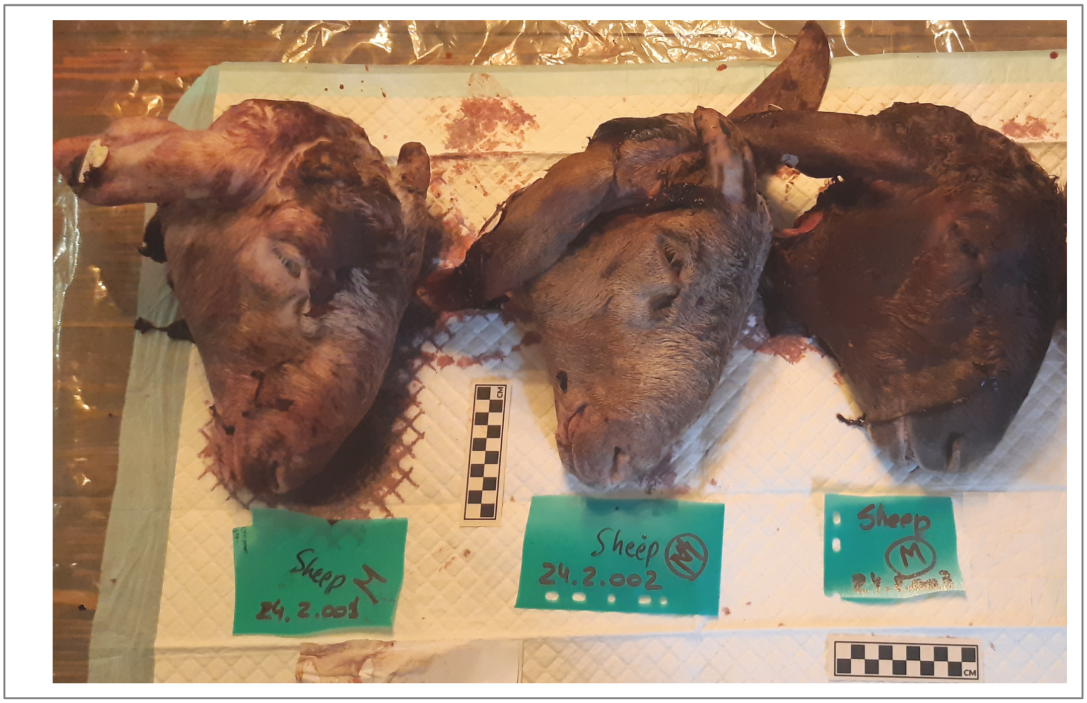

SI Figure 2: Three male *Ovis aries* (sheep) heads as received from Shefa-Amr Slaughterhouse, Israel.

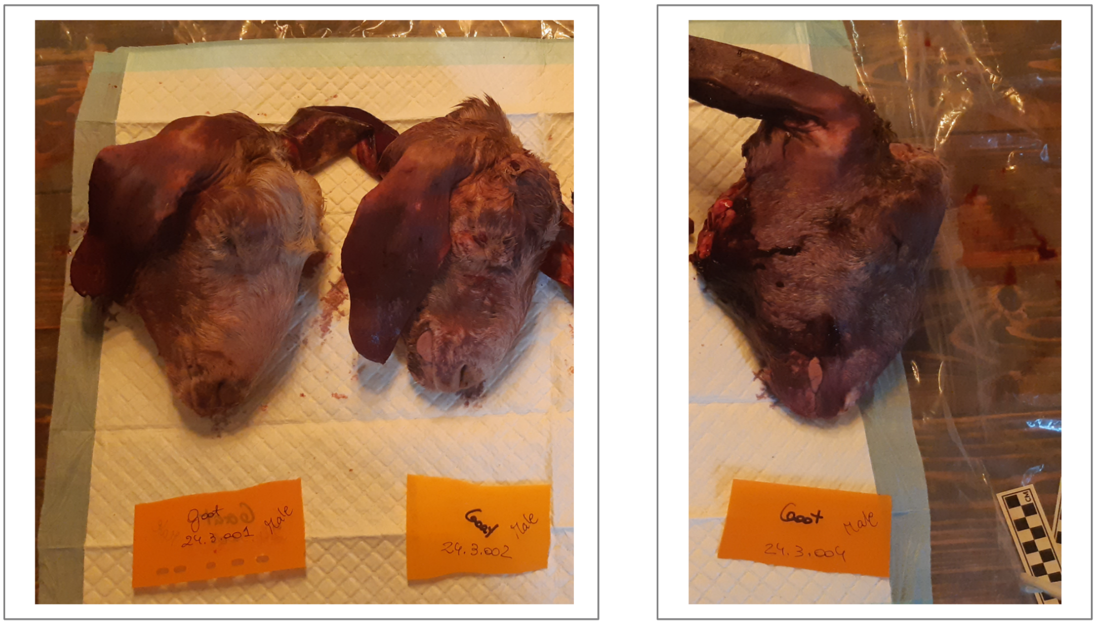

SI Figure 3: Three male *Capra hircus* (goat) heads as received from Shefa-Amr Slaughterhouse, Israel.

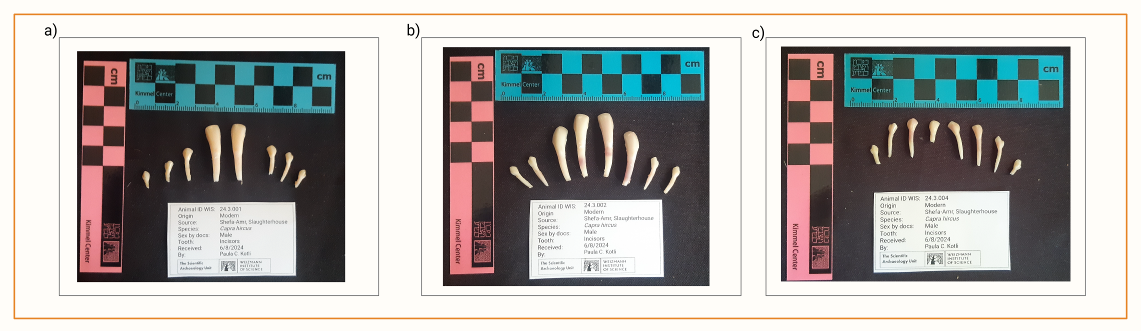

SI Figure 4: Female goat samples: a) WIS302, b) WIS303, c)WIS304 and WIS305

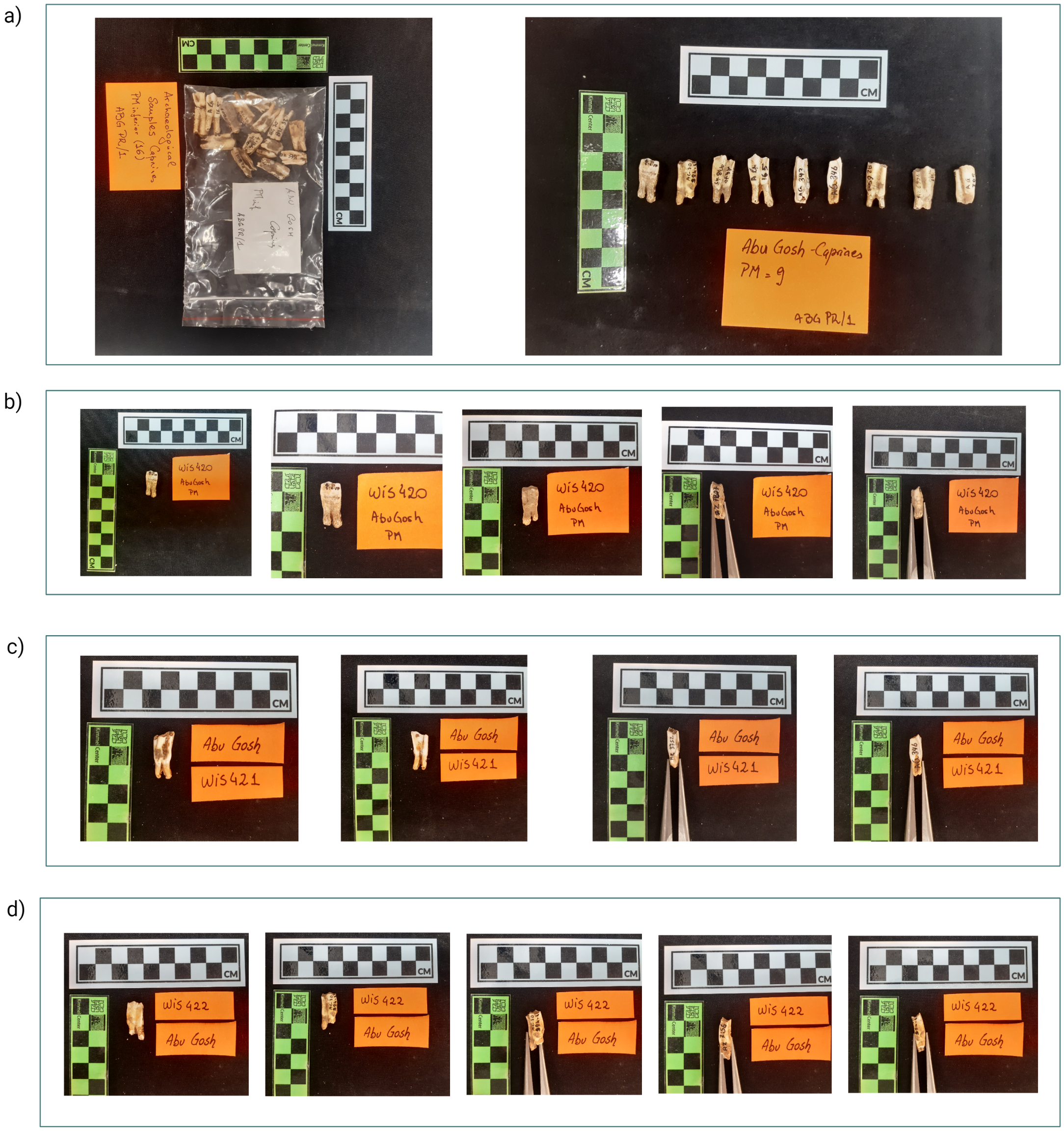

SI Figure 5: Abu Gosh selected caprine samples used in this research. a) Samples as received from Dr. Liora Kolska (author); b) to d) Samples WIS420 to 422.

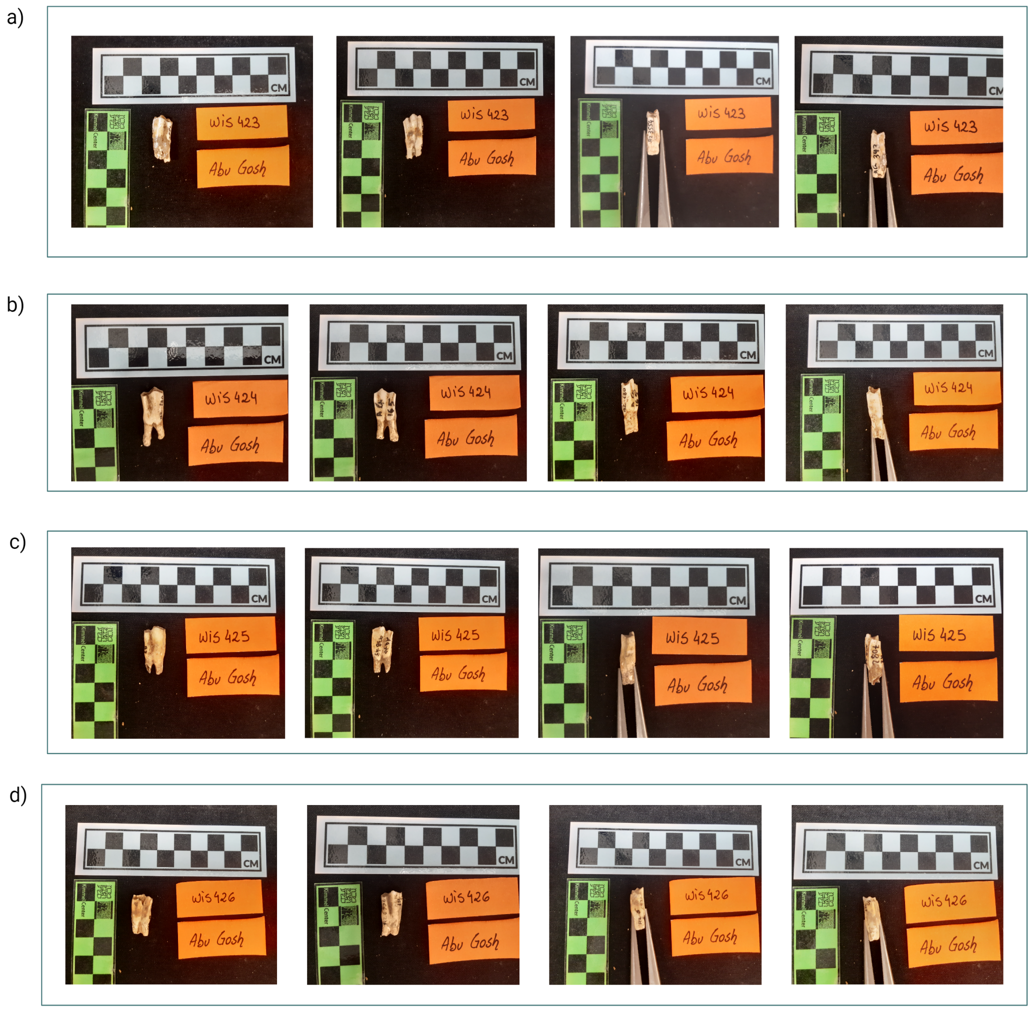

SI Figure 6: Abu Gosh selected caprine samples used in this research. a) to d) Samples WIS423 to 426.

a)

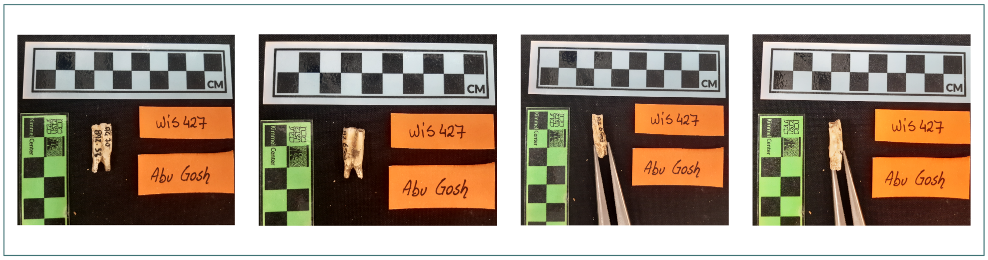

b)

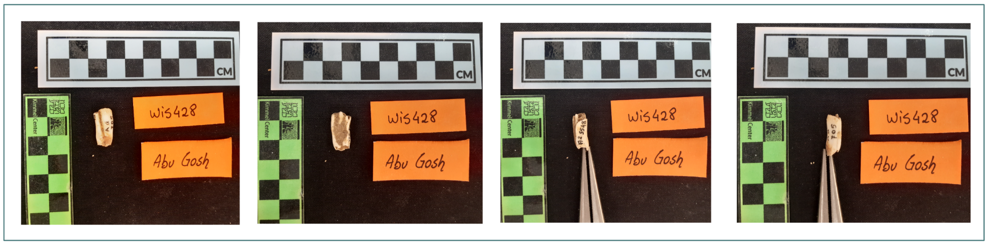

c)

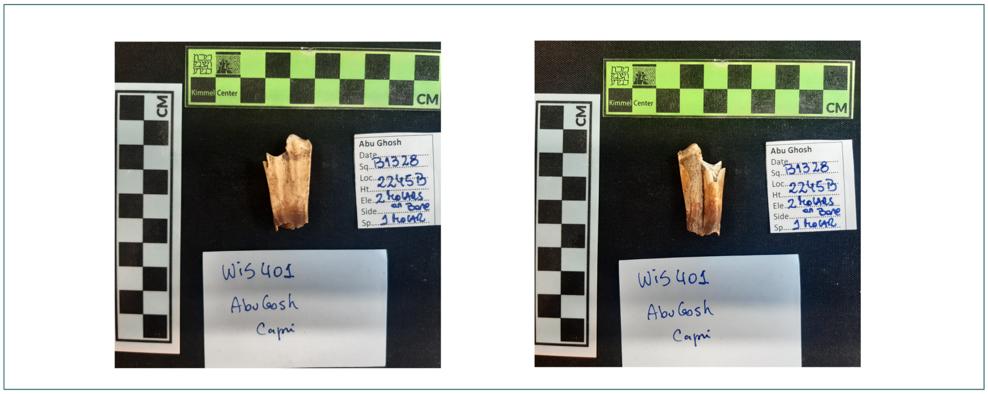

SI Figure 7: Abu Gosh selected caprine samples used in this research. a) WIS427 b) WIS428 c) Samples WIS429.

2 Teeth Before and After Mechanical Cleaning

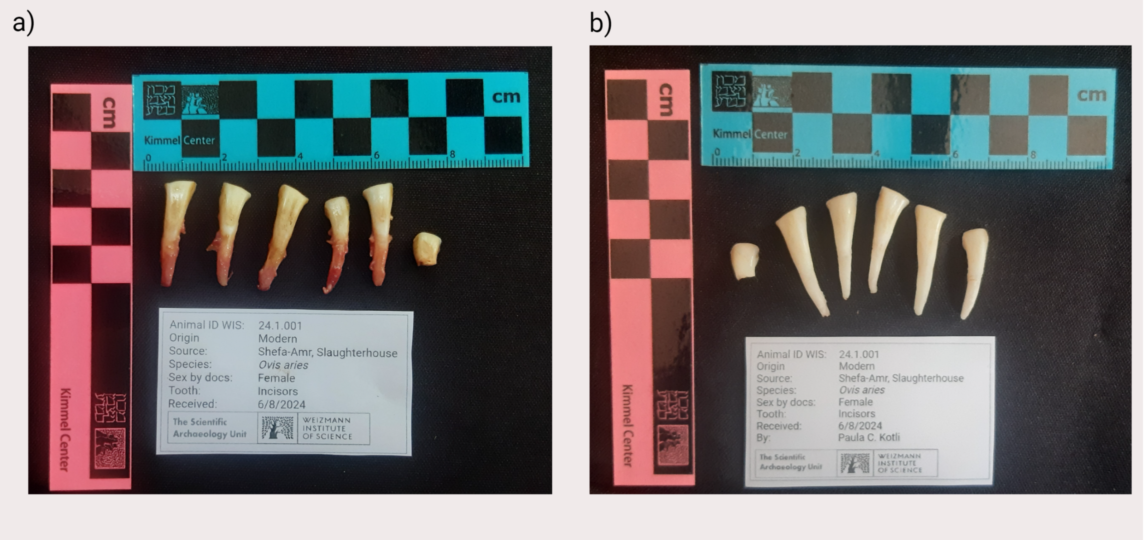

SI Figure 8: Incisor teeth for specimen ID 24.1.001 female sheep (example): (a) before extraction, and (b) after cleaning with cold water and a scalpel.

3 All tooth samples after cleaning

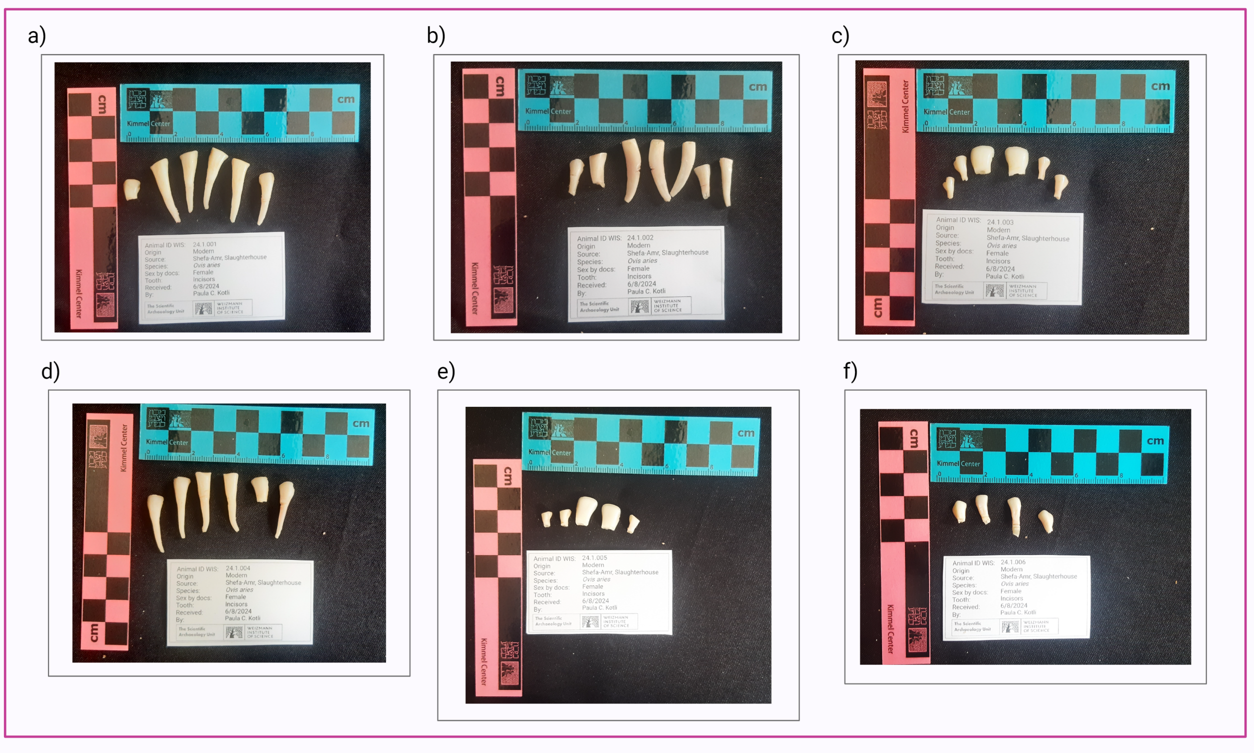

SI Figure 9: Six extracted sheep incisors after cleaning, animal IDs 24.1.001 to 24.1.006.

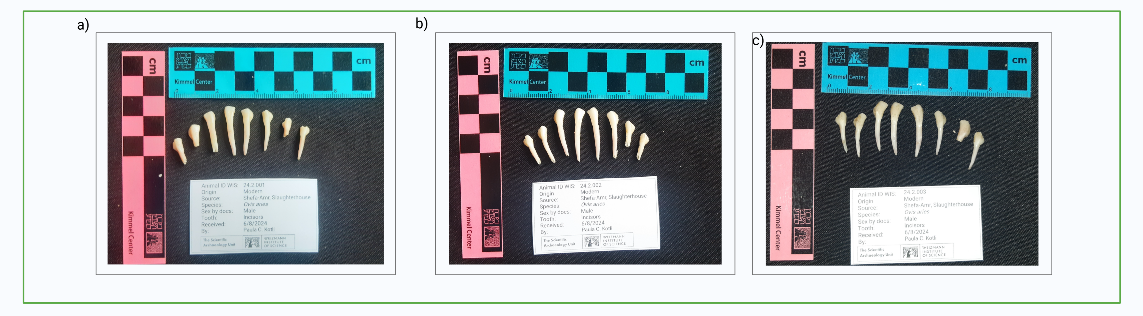

SI Figure 10: Three extracted sheep incisors after cleaning, animal IDs 24.2.001 to 24.2.003.

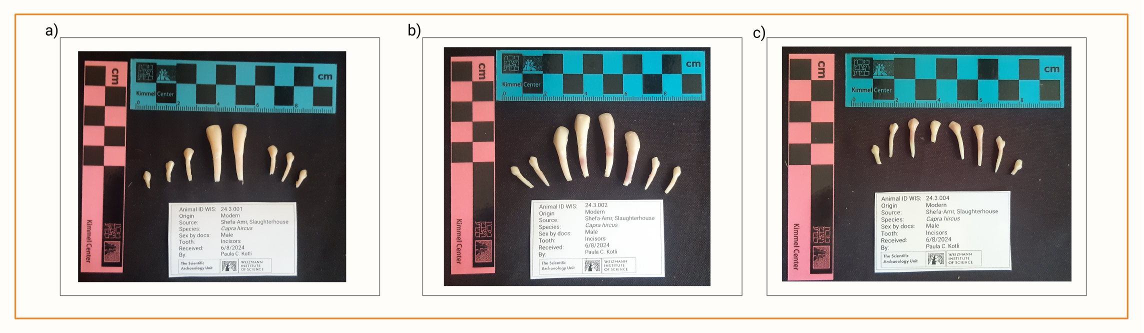

SI Figure 11: Three extracted goat incisors after cleaning, animal IDs 24.3.001, 24.3.002 and 24.3.004

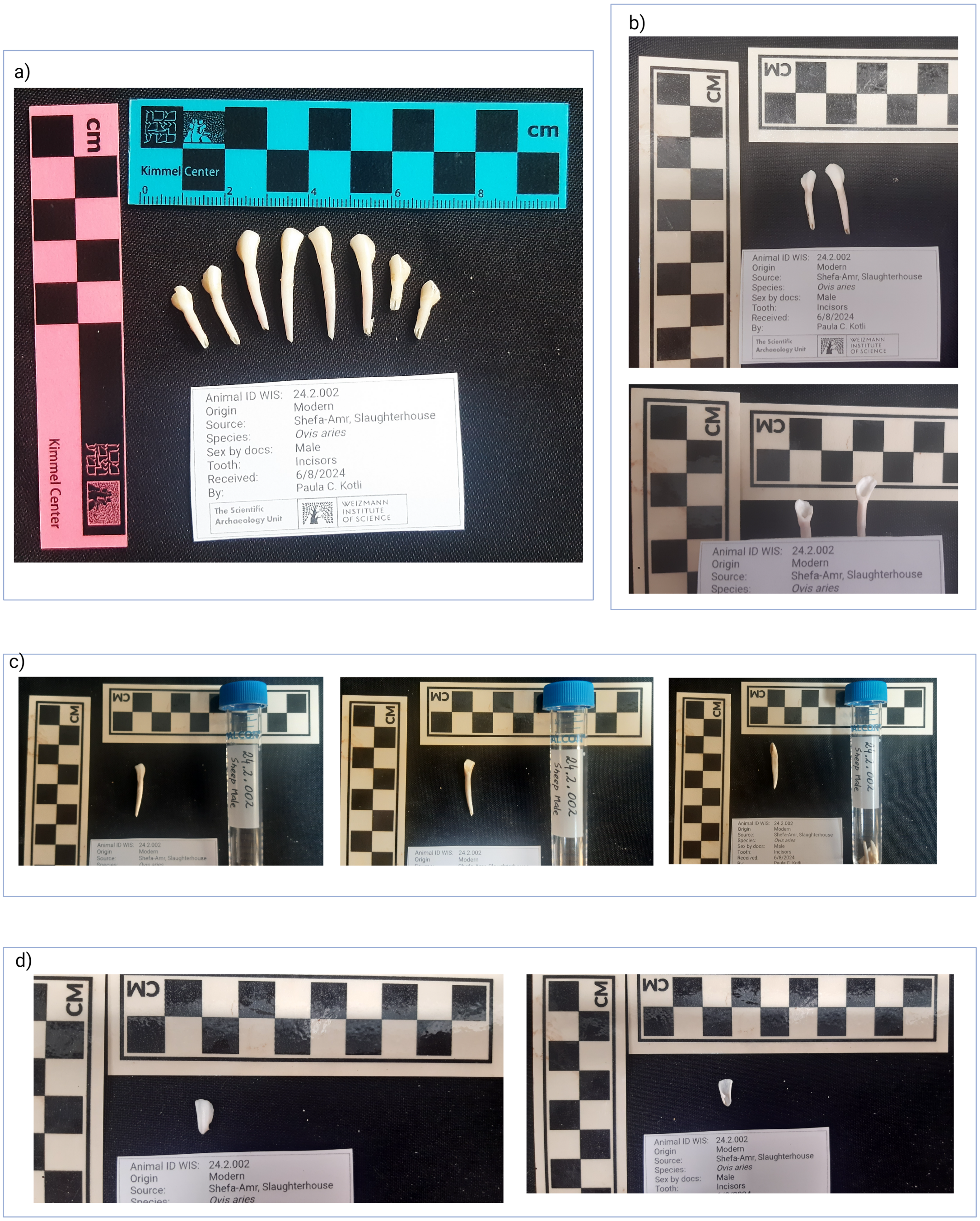

SI Figure 12: Enamel extraction process. a) all incisors extracted from animal 24.2.002; b) and c) after initial cleaning of dentine and d) enamel after cleaning and dentine removal, ready to use.

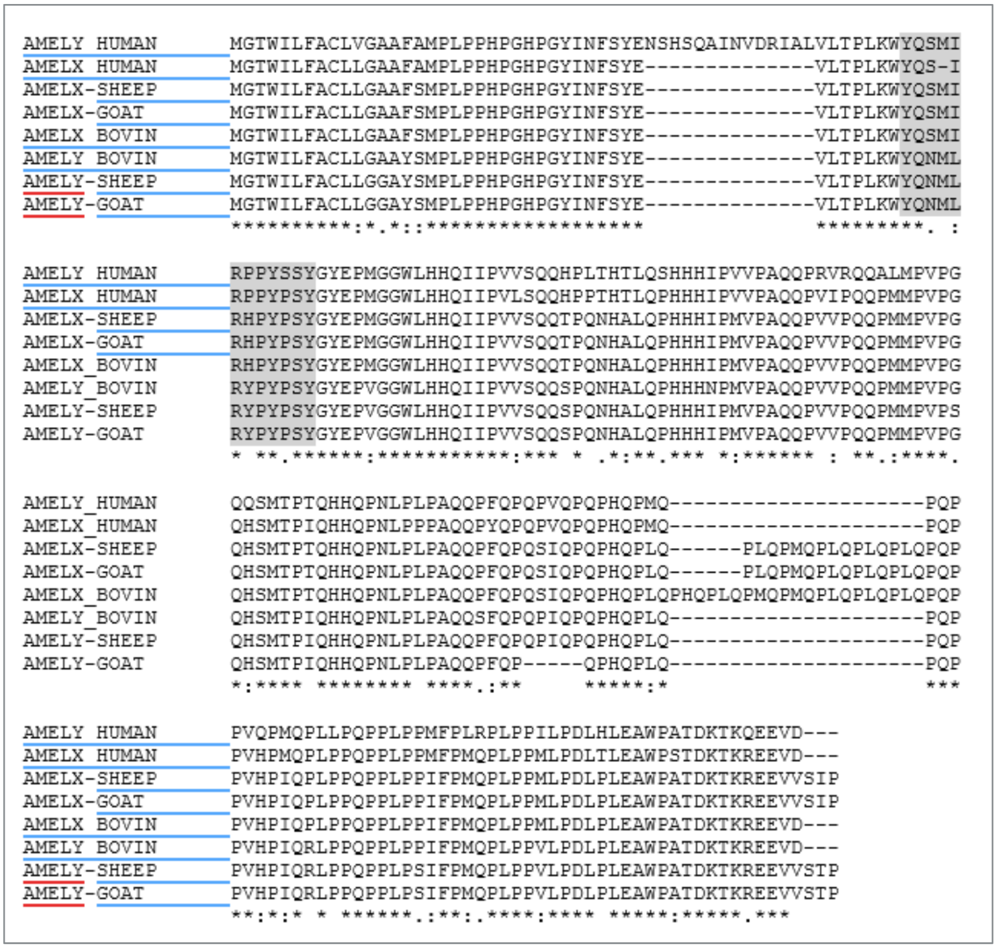

SI Figure 13: Alignment of human, cattle, sheep and goat AmelX/AmelY. In gray background, region shown in Figure 3.

```
AMELX_friesian     MGTWILFACLLGAAFSMPLPPHPGHPGYINFSYEVLTPLKWYQSMIRHPYPSYGYEPMGG
AMELX_kermani      MGTWILFACLLGAAFSMPLPPHPGHPGYINFSYEVLTPLKWYQSMIRHPYPSYGYEPMGG
AMELX_dorper       MGTWILFACLLGAAFSMPLPPHPGHPGYINFSYEVLTPLKWYQSMIRHPYPSYGYEPMGG
AMELX_romxdor      MGTWILFACLLGAAFSMPLPPHPGHPGYINFSYEVLTPLKWYQSMIRHPYPSYGYEPMGG
AMELX_hu           MGTWILFACLLGAAFSMPLPPHPGHPGYINFSYEVLTPLKWYQSMIRHPYPSYGYEPMGG
AMELY_friesian     MGTWILFACLLGGAYSMPLPPHPGHPGYINFSYEVLTPLKWYQNMLRYPYPSYGYEPVGG
AMELY_kermani      MGTWILFACLLGGAYSMPLPPHPGHPGYINFSYEVLTPLKWYQNMLRYPYPSYGYEPVGG
AMELY_dorper       MGTWILFACLLGGAYSMPLPPHPGHPGYINFSYEVLTPLKWYQNMLRYPYPSYGYEPVGG
AMELY_hu           MGTWILFACLLGGAYSMPLPPHPGHPGYINFSYEVLTPLKWYQNMLRYPYPSYGYEPVGG
AMELY_romxdor      MGTWILFACLLGGAYSMPLPPHPGHPGYINFSYEVLTPLKWYQNMLRYPYPSYGYEPVGG
                   ************.*:****************************.*:*:*********:**

AMELX_friesian     WLHHQIIPVVSQQTPQNHALQPHHHIPMVPAQQPVVPQQPMMPVPGQHSMTPTQHHQPNL
AMELX_kermani      WLHHQIIPVVSQQTPQNHALQPHHHIPMVPAQQPVVPQQPMMPVPGQHSMTPTQHHQPNL
AMELX_dorper       WLHHQIIPVVSQQTPQNHALQPHHHIPMVPAQQPVVPQQPMMPVPGQHSMTPTQHHQPNL
AMELX_romxdor      WLHHQIIPVVSQQTPQNHALQPHHHIPMVPAQQPVVPQQPMMPVPGQHSMTPTQHHQPNL
AMELX_hu           WLHHQIIPVVSQQTPQNHALQPHHHIPMVPAQQPVVPQQPMMPVPGQHSMTPTQHHQPNL
AMELY_friesian     WLHHQIIPVVSQQSPQNHALQPHHHIPMVPAQQPVVPQQPMMPVPSQHSMTPIQHHQPNL
AMELY_kermani      WLHHQIIPVVSQQSPQNHALQPHHHIPMVPAQQPVVPQQPMMPVPSQHSMTPIQHHQPNL
AMELY_dorper       WLHHQIIPVVSQQSPQNHALQPHHHIPMVPAQQPVVPQQPMMPVPSQHSMTPIQHHQPNL
AMELY_hu           WLHHQIIPVVSQQSPQNHALQPHHHIPMVPAQQPVVPQQPMMPVPSQHSMTPIQHHQPNL
AMELY_romxdor      WLHHQIIPVVSQQSPQNHALQPHHHIPMVPAQQPVVPQQPMMPVPGQHSMTPTQHHQPNL
                   *************:*******************************.****** *******

AMELX_friesian     PLPAQQPFQPQSIQPQPHQPLQPLQPMQPLQPLQPLQPQPPVHPIQPLPPQPPLPPIFPM
AMELX_kermani      PLPAQQPFQPQSIQPQPHQPLQPLQPMQPLQPLQPLQPQPPVHPIQPLPPQPPLPPIFPM
AMELX_dorper       PLPAQQPFQPQSIQPQPHQPLQPLQPMQPLQPLQPLQPQPPVHPIQPLPPQPPLPPIFPM
AMELX_romxdor      PLPAQQPFQPQSIQPQPHQPLQPLQPMQPLQPLQPLQPQPPVHPIQPLPPQPPLPPIFPM
AMELX_hu           PLPAQQPFQPQSIQPQPHQPLQPLQPMQPLQPLQPLQPQPPVHPIQPLPPQPPLPPIFPM
AMELY_friesian     PLPAQQPFQPQPIQPQPHQPLQ---------------PQPPVHPIQRLPPQPPLPSIFPM
AMELY_kermani      PLPAQQPFQPQPIQPQPHQPLQ---------------PQPPVHPIQRLPPQPPLPSIFPM
AMELY_dorper       PLPAQQPFQPQPIQPQPHQPLQ---------------PQPPVHPIQRLPPQPPLPSIFPM
AMELY_hu           PLPAQQPFQPQPIQPQPHQPLQ---------------PQPPVHPIQRLPPQPPLPSIFPM
AMELY_romxdor      PLPAQQPFQPQSIQPQPHQPLQ---------------PQPPVHPIQRLPPQPPLPSIFPM
                   ***********.**********               ********* ********.****

AMELX_friesian     QPLPPMLPDLPLEAWPATDKTKREEVVSIP
AMELX_kermani      QPLPPMLPDLPLEAWPATDKTKREEVVSIP
AMELX_dorper       QPLPPMLPDLPLEAWPATDKTKREEVVSIP
AMELX_romxdor      QPLPPMLPDLPLEAWPATDKTKREEVVSIP
AMELX_hu           QPLPPMLPDLPLEAWPATDKTKREEVVSIP
AMELY_friesian     QPLPPVLPDLPLEAWPATDKTKREEVVSTP
AMELY_kermani      QPLPPVLPDLPLEAWPATDKTKREEVVSTP
AMELY_dorper       QPLPPVLPDLPLEAWPATDKTKREEVVSTP
AMELY_hu           QPLPPVLPDLPLEAWPATDKTKREEVVSTP
AMELY_romxdor      QPLPPMLPDLPLEAWPATDKTKREEVVSTP
                   *****.********************** *
```

SI Figure 14: Alignment of five sheep species AmelX/AmelY Five different breeds: Friesian, Dorper (china), Hu, Kermani, F1: Romanov (mother) x Dorper (father) (from Kentucky, USA). The Romanov x dorper is showing crossing over in the last exon, though it is not clear if it is due to true cross-over, or to mistaken genome build. In Red, Y specific amino acids. In yellow background, putative cross-over residues. In light gray background, region of specific peptide.
